# Unravelling the mechanisms causing murepavadin resistance in *Pseudomonas aeruginosa*: lipopolysaccharide alterations and its consequences

**DOI:** 10.3389/fcimb.2024.1446626

**Published:** 2024-12-06

**Authors:** Marta Hernández-García, Raquel Barbero-Herranz, Natalia Bastón-Paz, María Díez-Aguilar, Eduardo López-Collazo, Francesc J. Márquez-Garrido, José María Hernández-Pérez, Fernando Baquero, Miquel B. Ekkelenkamp, Ad C. Fluit, Víctor Fuentes-Valverde, Miriam Moscoso, Germán Bou, Rosa del Campo, Rafael Cantón, José Avendaño-Ortiz

**Affiliations:** ^1^ Servicio de Microbiología, Hospital Universitario Ramón y Cajal, Instituto Ramón y Cajal de Investigación Sanitaria (IRYCIS), Madrid, Spain; ^2^ CIBER de Enfermedades Infecciosas, Instituto de Salud Carlos III, Madrid, Spain; ^3^ Servicio de Microbiología y Parasitología, Hospital Universitario La Princesa, Madrid, Spain; ^4^ CIBER de Enfermedades Respiratorias, Instituto de Salud Carlos III, Madrid, Spain; ^5^ Innate Immune Response Group, IdiPAZ, Madrid, Spain; ^6^ Bruker Microbiology & Infection Diagnostics, Bruker Española S.A., Madrid, Spain; ^7^ Plataforma de Proteómica y Metabolómica, Instituto de Investigación Germans Trias i Pujol, Badalona, Spain; ^8^ CIBER de Epidemiología y Salud Pública, Instituto de Salud Carlos III, Madrid, Spain; ^9^ Department of Medical Microbiology, University Medical Center Utrecht, Utrecht, Netherlands; ^10^ Department of Microbiology, University Hospital A Coruña (CHUAC)-Biomedical Research Institute A Coruña (INIBIC), A Coruña, Spain

**Keywords:** murepavadin, colistin, antibiotic resistance, host-derived antimicrobial peptides, monocytes, innate immune system

## Abstract

**Introduction:**

Murepavadin is an antimicrobial peptide (AMP) in clinical development that selectively targets *Pseudomonas aeruginosa* LptD and whose resistance profile remains unknown. We aimed to explore genomic modifications and consequences underlying murepavadin and/or colistin susceptibility.

**Methods:**

To define genomic mechanisms underlying resistance, we performed two approaches: 1) a genome-wide association study (GWAS) in a *P. aeruginosa* clinical collection (n=496), considering >0.25 mg/L as tentative cut-off of murepavadin acquired resistance; 2) a paired genomic comparison in a subset of 5 isolates and their isogenic murepavadin-resistant mutants obtained *in vitro*. Lipid-A composition, immunogenicity and cathelicidin and indolicidin effects on bacterial growth were also tested in this last subset of isolates. Murepavadin MICs were determined in Δ*lpxL1* and Δ*lpxL2* knock-out mutants obtained from a auxotroph PAO1 derivative.

**Results:**

GWAS revealed a missense variant (A→G p.Thr260Ala in the *hisJ* gene) associated with murepavadin resistance although both resistant and susceptible strains harbored it (21% and 12% respectively, OR=1.92, p=0.012 in χ² test). Among the isolate subset, murepavadin-resistant mutants with deletions in *lpxL1* and *lpxL2* genes showed lower abundance of hexa-acylated lipid-A (m/z 1616, 1632). 4-aminoarabinose addition was found only in colistin-resistant isolates but not in the other ones, irrespective of murepavadin susceptibility. Accordingly, Δ*lpxL1* and Δ*lpxL2* mutants exhibited higher murepavadin MICs than parental PAO1 auxotroph strain (2 and 4 *vs* 0.5 mg/L respectively). Lipopolysaccharide from murepavadin-resistant mutants triggered lower inflammatory responses in human monocytes. Those with *lpxL* mutations and hexa-acylated lipid-A loss also exhibited greater growth reduction when exposed to host-derived AMPs cathelicidin and indolicidin.

**Discussion:**

High murepavadin-resistance seems to be linked to *lpxL1* and *lpxL2* mutations and lower hexa-acylated lipid-A, corresponding to lower inflammatory induction and higher susceptibility to host-derived AMPs. Although GWAS identified one variant associated with the murepavadin-resistant phenotype, data revealed that there was no unique single genetic event underlying this phenotype. Our study provides insight into the mechanisms underlying murepavadin susceptibility.

## 1 Introduction

Antimicrobial peptides (AMPs) are small peptide-derived molecules (12-60 amino acids) with bactericidal activity (Huan et al., 2020; Talapko et al., 2022) that can be produced by host immune cells, such as cathelicidin or defensins, but also by microorganisms; colistin, vancomycin, and daptomycin are examples of the latter (Wang and Mechesso, 2022). AMPs represent a promising strategy for the discovery of new antimicrobial drugs (Mba and Nweze, 2022). Murepavadin (POL7080; Polyphor Ltd) is a new peptidomimetic, initially developed for systemic administration and currently being positioned for inhaled therapy (NCT03582007 and NCT03409679). Its target is the protein transporter, LptD, blocking lipopolysaccharide transport by the bacterium (Martin-Loeches et al., 2018). Murepavadin has notable and almost exclusive activity against *Pseudomonas aeruginosa*, including in biofilm (Díez-Aguilar et al., 2021), and it is considered an ecologic antibiotic for its lack of effect on the commensal microbiota. In addition, this antibiotic promotes the immune response, facilitating bacterial clearance (Amponnawarat et al., 2021). The mechanism underlying the decrease of susceptibility to murepavadin is not completely characterized, although previous studies have reported a tandem duplication of 6 residues in the periplasmic part of LptD (Andolina et al., 2018) and mutations in lipopolysaccharide/lipid A genes (Díez-Aguilar et al., 2021; Ghassani et al., 2024).

On the other hand, the polymyxin, colistin, is a cationic peptide that has been used in the last 2 decades as a last resort for severe infections caused by multidrug-resistant microorganisms, including *P. aeruginosa*. This positively charged antibiotic interacts electrostatically with the negatively charged lipid A phosphate group, causing destabilization of the outer membrane, pore formation, increased permeability, and cell lysis. In *Enterobacterales*, colistin resistance has been mainly associated with dissemination of *mcr* genes harbored in plasmids, but this is not a common mechanism in *P. aeruginosa* (Snesrud et al., 2018). In contrast, lipopolysaccharide modification and negative charge reduction by overproduction of 4-amino-L-arabinose (L-Ara4N) is one of the major causes of colistin resistance in *P. aeruginosa* (Lo Sciuto et al., 2020). The synthesis and the transport of L-Ara4N molecules are encoded by the large *arn*BCADTEF-UgD operon, simplified as *arn*, which has a complex regulatory network (Barrow and Kwon, 2009; Nowicki et al., 2015; Jochumsen et al., 2016; Bolard et al., 2019).

The ability to predict antimicrobial resistance using bioinformatics tools and bacterial genetic content has gained attention. In previous studies, we and others used whole genome sequencing (WGS) and single nucleotide polymorphisms (SNPs) analysis of specific relevant genes to explore their association with murepavadin resistance (Díez-Aguilar et al., 2021; Ghassani et al., 2024). Here, we propose an untargeted strategy by performing a genome-wide association study (GWAS), a recently successful approach in the study of genotype-phenotype associations in prokaryotes (Falush, 2016; Diaz Caballero et al., 2018; Bokma et al., 2021; Young et al., 2021). Furthermore, lipid A structural modifications, immunogenicity and the possible cross-resistance to host-derived AMPs were performed in a subset of murepavadin-resistant mutants previously obtained *in vitro* (Ekkelenkamp et al., 2020; Díez-Aguilar et al., 2021).

## 2 Material and methods

### 2.1 Bacterial strains, antibiotic susceptibility, and whole genome sequencing

A collection of 496 *P. aeruginosa* isolates previously obtained during the “Inhaled antibiotics in bronchiectasis and cystic fibrosis” (iABC) project from the Netherlands (n=240), Spain (n=139), Northern Ireland (n=94), and Australia (n=23) (https://www.imi.europa.eu/projects-results/project-factsheets/iabc) were used (Ekkelenkamp et al., 2020). Isolates were obtained from the airway of patients with cystic fibrosis (n=399), chronic obstructive pulmonary disease (n=29), bronchiectasis (n=30), or other respiratory pathologies (n=38). A single isolate per patient was considered. Susceptibility to murepavadin and colistin was determined by ISO-broth microdilution, following European Committee on Antimicrobial Susceptibility Testing (EUCAST) recommendations and interpretation (EUCAST v12.0, 2022) (The European Committee on Antimicrobial Susceptibility Testing, 2022). Colistin minimum inhibitory concentration (MIC) of >4 mg/L and murepavadin MIC of >0.25 mg/L were considered as a cut-off and tentative cut-off, respectively, to define resistant isolates (Ekkelenkamp et al., 2020, 2022; Díez-Aguilar et al., 2021; The European Committee on Antimicrobial Susceptibility Testing, 2022). WGS was obtained in an Illumina MiSeq or NextSeq platform (Illumina, San Diego, CA, USA), employing the Nextera XT library prep kit (Illumina) with 2x150 paired-end reads. All reads were trimmed with seqtk trimfq version 1.3 with an error rate threshold of 0.001. Sequence reads from each isolate were assembled with SPAdes v3.11.1. Sequences were deposited at the European Nucleotide Archive under BioProject ID: PRJNA527751 and PRJNA530912. Bacterial identification was confirmed by k-mer-based classification using Kraken (v1.1.1). All draft genomes were annotated by Prokka (v1.14.6). Resistance genes were screened with Abricate (v0.8) (ARG-ANNOT, CARD and ResFinder databases; threshold, 95% identity; 90% coverage). The Mash v. 2.3 and iTOL applications were employed to generate and trace a similarity tree based on a neighbor-joining algorithm.

### 2.2 Variant calling

The allelic variants in the whole genome were determined in a first stage, with the second analysis restricted to a subset of genes previously associated with colistin and/or murepavadin resistance in *P. aeruginosa* (Díez-Aguilar et al., 2021; Hernández-García et al., 2021). SNPs and small insertions or deletions were extracted with Snippy (v4.6.0), using the *P. aeruginosa* PAO1 (NC_002516.2) genome as reference. Synonymous variants were excluded for further analysis.

### 2.3 Genome-wide association study analysis

An appropriate correction considering the clonal population structure was applied by pyseer (v1.3.10) according to the fixed effects model of software documentation, with the binary phenotype as susceptible or resistant. Genes, regions, or SNPs with a minor allele frequency of <0.01 were not tested. Performing sequence element enrichment analysis in pyseer can accurately detect associations with phenotype (in our case, antibiotic resistance) caused by SNPs in coding regions. The screening of allelic variants was performed independent of colistin or murepavadin MIC values. The clonal population structure was estimated/inferred employing a pairwise distance matrix generated with mash (v.3.2) that measures genetic similarity as the co-variance between the individual isolate genetic variant vectors. Bonferroni correction was applied in the statistical analysis, only considering results with *p* values <0.5.

### 2.4 Lipid A characterization

Lipid A characterization was performed on a subset of 5 P*. aeruginosa* isolate pairs, including 4 clinical isolates and the reference strain PAO1, and their corresponding murepavadin-resistant mutant derivatives previously reported (Díez-Aguilar et al., 2021). The selection of the isolates was based on their colistin MIC values, their ST lineage, and the growth mode (**Supplementary Table S1**). Lipid A was extracted with the MBT Lipid Xtract Kit (Bruker Daltonics GmbH) following the manufacturer’s instructions. Briefly, a 1-µL inoculation loop of bacteria from a fresh overnight culture of the bacterial strains grown on a Columbia blood agar plate was subjected to hydrolysis at 90°C for 10 min. The dry pellets were washed and left 2 min at 90°C for evaporation. Then, the pellet was resuspended in Lipid Xtract Matrix, and 2 µL were spiked into an MTP 384 target plate polished steel BC (Bruker Daltonics GmbH). Mass spectrometry runs were performed by matrix-assisted laser desorption ionization–time of flight (MALDI-TOF) in an Autoflex maX spectrometer (Bruker Daltonics GmbH), in linear negative-ion mode. Spectra were analyzed with Clover MS Data Analysis Software v1.6.4 (CLOVER Bioanalytical Software S.L.).

### 2.5 Murepavadin MIC values determination of *lpxL1* and *lpxL2* auxotroph PAO1 mutants

The bacterial PAO1 mutant strains tested included PAO1 auxotroph mutants obtained in previous studies (Cabral et al., 2020; Fuentes-Valverde et al., 2022). The parental auxotroph PAO1 strain was a triple mutant including in-frame deletions of three genes (Δ*murI*, Δ*alr* and Δ*dadX*) making it auxotroph for D-glutamate and D-alanine (Cabral et al., 2020; Fuentes-Valverde et al., 2022). In this auxotrophic PAO1- strain, an additional deletion of *lpxL1* and *lpxL2* were used to form the Δ*lpxL1* and Δ*lpxL2* strains respectively. The MIC values of murepavadin in these strains was determined by the microdilution method in 96-well U-bottom plates (Corning). Briefly, a 0.5 McFarland scale inoculum was prepared in a sterile saline solution for each strain. This initial suspension was 1:100 diluted in LB broth supplemented with 8 mM of D-glutamate and 6 mM of D-alanine. Murepavadin concentrations tested ranged from 0.015 to 32 mg/L. After an overnight incubation at 37°C, the wells were examined for visible bacterial growth. The lowest concentration of murepavadin that prevented visible bacterial growth was defined as the MIC values. Experiment was repeated twice in independent experiments.

### 2.6 Lipopolysaccharide isolation and immunogenicity assay

Lipopolysaccharide was isolated using a lipopolysaccharide isolation kit (Sigma-Aldrich) according to the manufacturer’s protocol. Briefly, the 10 isolates (the selected 4 cystic fibrosis isolates plus PAO1 and their relative mutants) were grown overnight on Luria-Bertani (LB) medium agar plates (Difco). 200 mg of bacteria were taken with sterile 10 µL loops and resuspended in 10 mL of cold sterile PBS. The suspensions were centrifugated (2,500 x g for 10 min) to pellet the bacteria resuspended in 2 mL of lysis buffer and sonicated (3x30 s in continuous pulse). Then they were incubated on ice for 10 min washed (2,500 x g for 10 min, 4°C) and the lysate were transferred to a clean tube. Finally, proteinase K was added to a final concentration of 100 mg/L, heated 60 min at 60 °C then centrifuged (2,500 x g for 10 min, 4°C) and supernatant were transferred to a clean tube. Lipopolysaccharide in these supernatants was quantified with a total carbohydrate assay kit (Sigma-Aldrich). Blood from 8 healthy adult volunteers was obtained from the Transfusion Center of the Community of Madrid (n=4 males and n=4 females; mean age ± SD, 52.4 ± 8.2). Peripheral blood mononuclear cells were isolated by Ficoll-Paque Plus (Cytiva) gradient. Cells were counted, and monocyte percentages were checked by flow cytometry (BD FACSCalibur). The monocyte population was enriched by adherence protocol with 1 h of culture in serum-free Roswell Park Memorial Institute (RPMI) medium as previously described (del Fresno et al., 2009; Avendaño-Ortiz et al., 2019). After this period, the non-adherent cells were removed and the adherent cells were washed three times with PBS, and finally cultured with RPMI-10% fetal bovine serum medium. An average of monocyte purity percentage of 55.6 ± 10.4 defined by CD14+ monocytes in the adherent cells were obtained. Cells were stimulated with 10 mg/L lipopolysaccharide for 16 h in a humidified 5% CO_2_ atmosphere at 37°C. A commercial LPS from *Escherichia coli* O111:B4 (Sigma-Aldrich, cat. number L2630) were used as positive control. Cytokine production in cell culture supernatants was determined by flow cytometry (BD FACSCalibur), employing the LEGENDplex™ Human Essential Immune Response Panel. Cells were labeled with CD14-APC antibody (Immunostep) and apoptosis on gated CD14+ cells were determined by flow cytometry (BD FACSCalibur) using FITC Annexin V Apoptosis Detection Kit (Immunostep) following manufacturer’s instructions.

### 2.7 Cross-resistance with human antimicrobial peptides

Starting from an overnight culture on Columbia blood agar, a bacterial inoculum was adjusted to 0.5 McFarland diluted 1:100 (v/v) in Mueller Hinton II Broth (Liofilchem^®^). Based on previous reports in *P. aeruginosa* growth inhibition, a concentration of 128 mg/L for cathelicidin and indolicidin was chosen (Gooderham et al., 2008; Mohammed et al., 2019; Xiao et al., 2022). Thus, the effects on bacterial growth of 128 mg/L cathelicidin and 128 mg/L indolicidin (Anaspec, Fremont, CA, USA) were determined by incubating the bacterial suspensions with the two peptides separately, in 96-microwell plates at 30°C with orbital shaking. Cell density was monitored at OD600 every 15 min with a Synergy HTX (BioTek).

## 3 Results

### 3.1 Genomic clinical isolates characteristics

**Figure 1:**
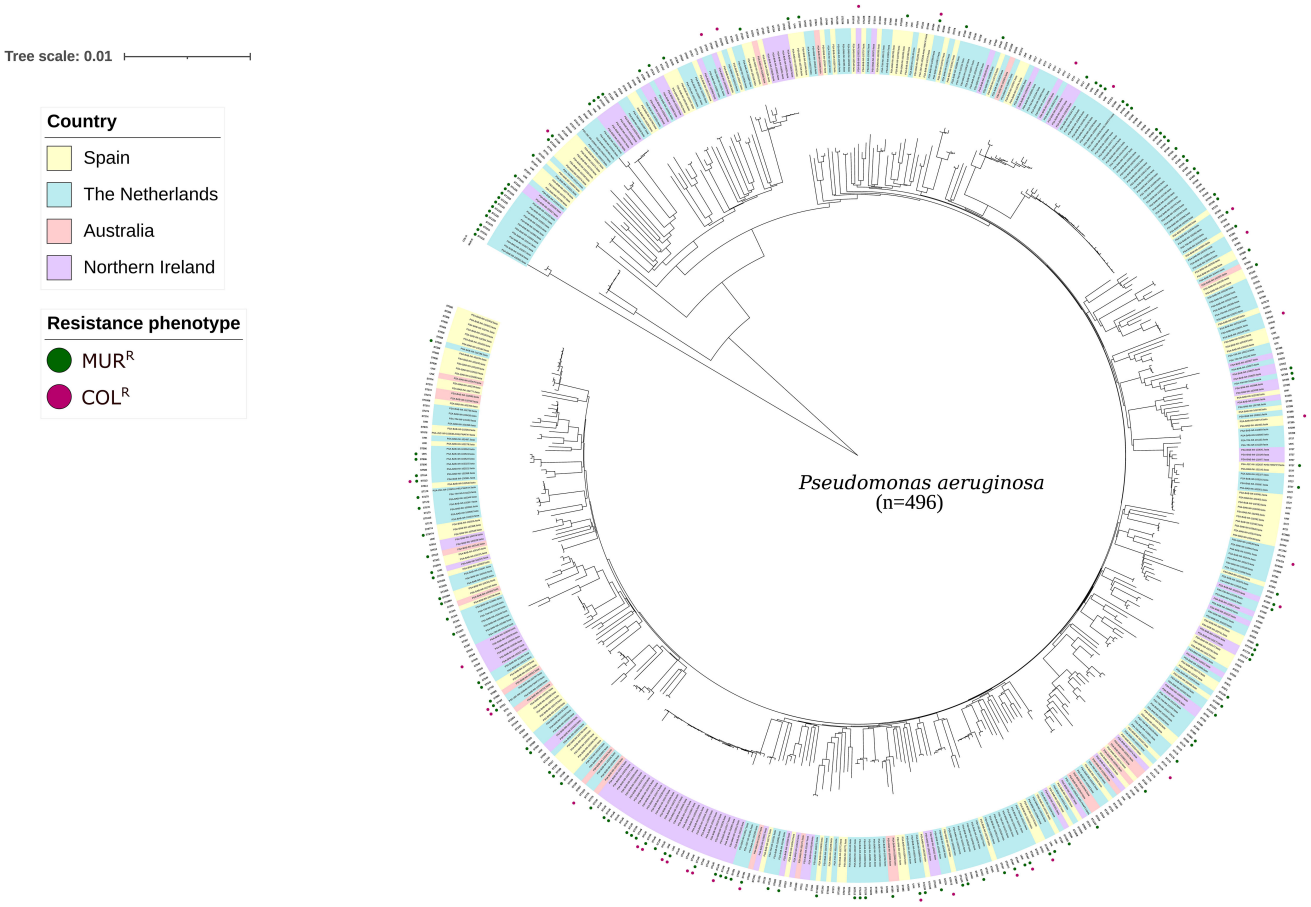
Similarity tree iTOL construction with the whole genome of the 496 *P. aeruginosa* isolates included in bioinformatic analysis. Legend differentiates the country of origin and their phenotypic susceptibility to murepavadin and colistin. MUR^R^, murepavadin resistant (MIC >0.25 mg/L) and COL^R^, colistin resistant (MIC >4mg/L).

A collection of 496 P*. aeruginosa* clinical isolates from patients with cystic fibrosis or other respiratory pathologies were included in the analysis. Up to 348 (70.2%) were susceptible to both colistin and murepavadin, 15 (3.0%) colistin-resistant and murepavadin-susceptible, 116 (23.4%) murepavadin-resistant and colistin-susceptible, and 17 (3.4%) were resistant to both antibiotics. Five clusters were identified (**Figure 1**). A high genetic variability was observed in our collection, including 179 sequence types (STs). Of note: 42 (8.5%) isolates could not be assigned to any ST. Overall, 20.9% (94/450) of the isolates yielded unique STs, the most prevalent being ST406 (n=30, 6.7%), followed by ST146 (n=27, 6%). Only 3 STs (ST155, ST179 and ST253) were represented in the 4 countries.

The average size of the *de novo* assembled genomes was 6.4 Mb, with a G + C content of 66.3%. All isolates resistant and susceptible to colistin carried the *arnA* gene, without other genes previously associated with colistin resistance. The pangenome of the 496 isolates included have 40,188 different orthologous genes, the core genome (genes in >95% of isolates) had 3,912 clusters (9.73% of all genes), and the accessory genome (genes in <15% of strains) had 2,629 clusters (6.54% of all genes).

### 3.2 Genome-wide association study analysis

**Figure 2:**
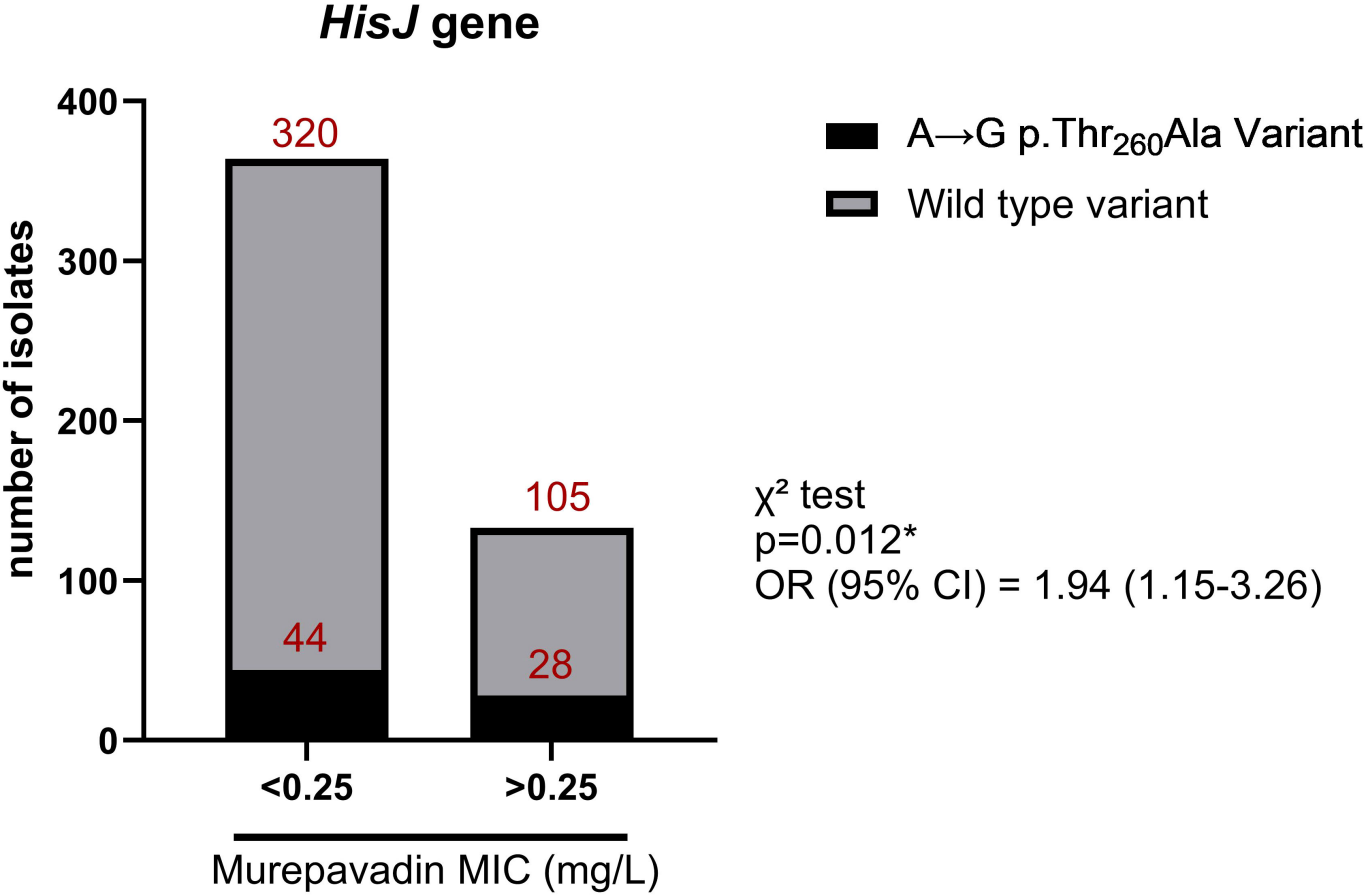
Number of *P. aeruginosa* isolates harboring wild type or A→G p.Thr_260_Ala variant according to their susceptibility to murepavadin. OR, odds ratio; *p-value < 0.05 in Chi-square (χ2) test.

An independent analysis was conducted for each antibiotic, excluding loci with a burden frequency below 0.01 and adjusting for population structure. Out of 777,515 allele variants, 102,008 non-synonymous were ultimately considered (**Supplementary Figure S1**). For colistin resistance, no allelic variants reached statistical significance although notable findings included a missense variant (C>T p.Asp921Asn) in the *recC* gene (p=2.25×10^-5^) and a variant in an intragenic region (p=1.97×10^-5^) (**Supplementary Table S2**). In the analysis of murepavadin resistance, a missense variant (A>G p.Thr260Ala) in the *hisJ* gene was significantly associated (p=7.77×10^-8^, OR=14.3, **Supplementary Table S3**) but it was not relevant for colistin resistance (p=0.28). This mutation was found in 21% of murepavadin-resistant strains, although it was also observed in 12% of the susceptible strains (OR=1.92, p=0.012 in χ² test) (**Figure 2**). A final GWAS was performed to identify variants associated with resistance to both colistin and murepavadin by comparing 17 isolates resistant to both antibiotics with the other 349 isolates. No significant variants were identified after Bonferroni correction (p=6.5×10^-7^). The highest associations were observed in intergenic regions, a protein of unknown function, and the *YhjE* and *YofA3* genes (**Supplementary Table S4**).

### 3.3 Loss of lipid A acyl chains and *lpxL1* and *lpxL2* variation in murepavadin-resistant mutants

**Figure 3:**
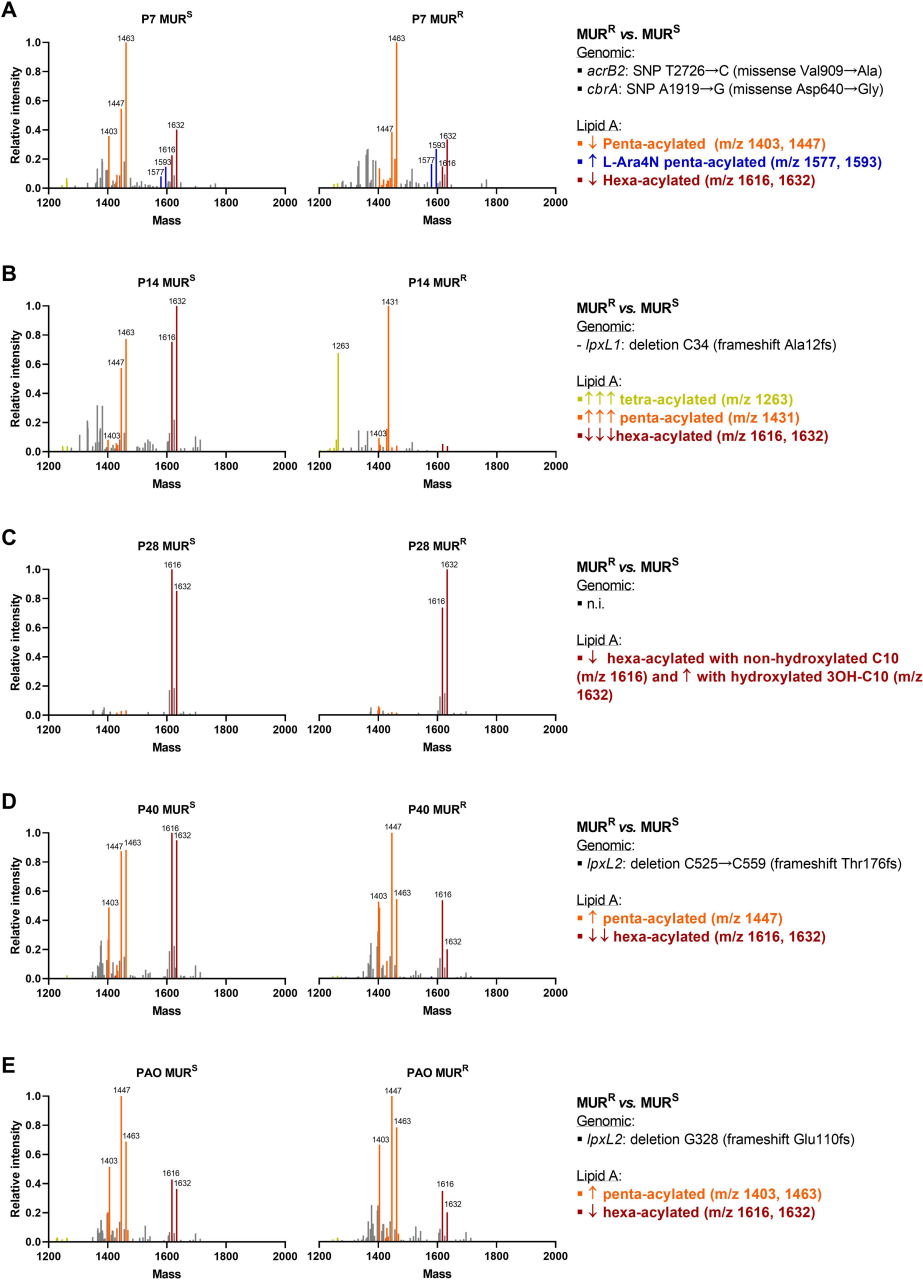
Lipid A profiles in the murepavadin resistant mutants (MUR^R^) and murepavadin susceptible parental strains (MUR^S^) of P7 **(A)**, P14 **(B)**, P28 **(C)**, P40 **(D)** and PAO **(E)** strain pairs. Mass spectrum expressed in relative intensity against maximum intensity peaks of the 10 *P. aeruginosa* isolates. Peaks corresponding to tetra-acylated lipid A are in yellow, penta-acylated lipid A in orange, hexa-acylated lipid A in red and L-Ara4N modified lipid A in blue. n.i., not identified.

Since we did not identify any specific single genetic event that contributes to murepavadin resistance in the clinical isolate collection, we decided to study the mechanisms behind the resistance in *in vitro* obtained murepavadin-resistant mutants. In our previous study we obtained and sequenced murepavadin-resistant mutants from 4 *P. aeruginosa* clinical isolates susceptible to murepavadin (description in **Supplementary Table S1**) (Díez-Aguilar et al., 2021). The lipid A profiles of these *P. aeruginosa* isolates were characterized by hexa-acylated lipid A as the most abundant forms in 3 out of the 4 clinical isolates, while PAO1 exhibited a dominance of penta-acylated m/z 1447. Proposed lipid A structures are shown in **Supplementary Figure S2**. Apart from isolate P28, which exhibited the same hexa-acylated ions (m/z 1616 and 1632) in both susceptible and resistant strains, the murepavadin-resistant mutants were characterized by a decrease of the hexa-acylated forms, along with an increase of penta-acylated and/or tetra-acylated forms. This phenomenon was especially pronounced in P14 and P40 isolates, those that also carried allelic variants in the *lpxL1* and *lpxL2* genes (**Figure 3**). Note that these genes encode acyltransferases involved in lipid A biogenesis (Hittle et al., 2015). The addition of L-Ara4N or pEtN to lipid A was only detected in the P7 isolate, which was the unique isolate with colistin resistance. As three of the five murepavadin-resistant mutants exhibited *lpxL1* and *lpxL2* deletions compared to their parental, we move to test if these two genes could be involved in murepavadin resistance. We found that the Δ*lpxL1* and Δ*lpxL2* strains exhibited MICs to murepavadin that were 4 to 8 folds higher than the auxotrophic PAO1 parental strain (**Table 1**).

**Table 1:** Murepavadin MIC values of PAO1 and auxotroph derivative mutants.

Strain	Murepavadin MIC (mg/L)	Murepavadin MIC fold increase
PAO1	0.5	1^#^
Auxotroph PAO1	0.5	1^*^
Auxotroph Δ*lpxL1* PAO1	2	4^*^,^#^
Auxotroph Δ*lpxL2* PAO1	4	8^*^,^#^

*, versus PAO1 strain; ^#^, versus PAO1 auxotrophic strain.

### 3.4 Murepavadin-resistant mutants induce a lesser inflammatory response

**Figure 4:**
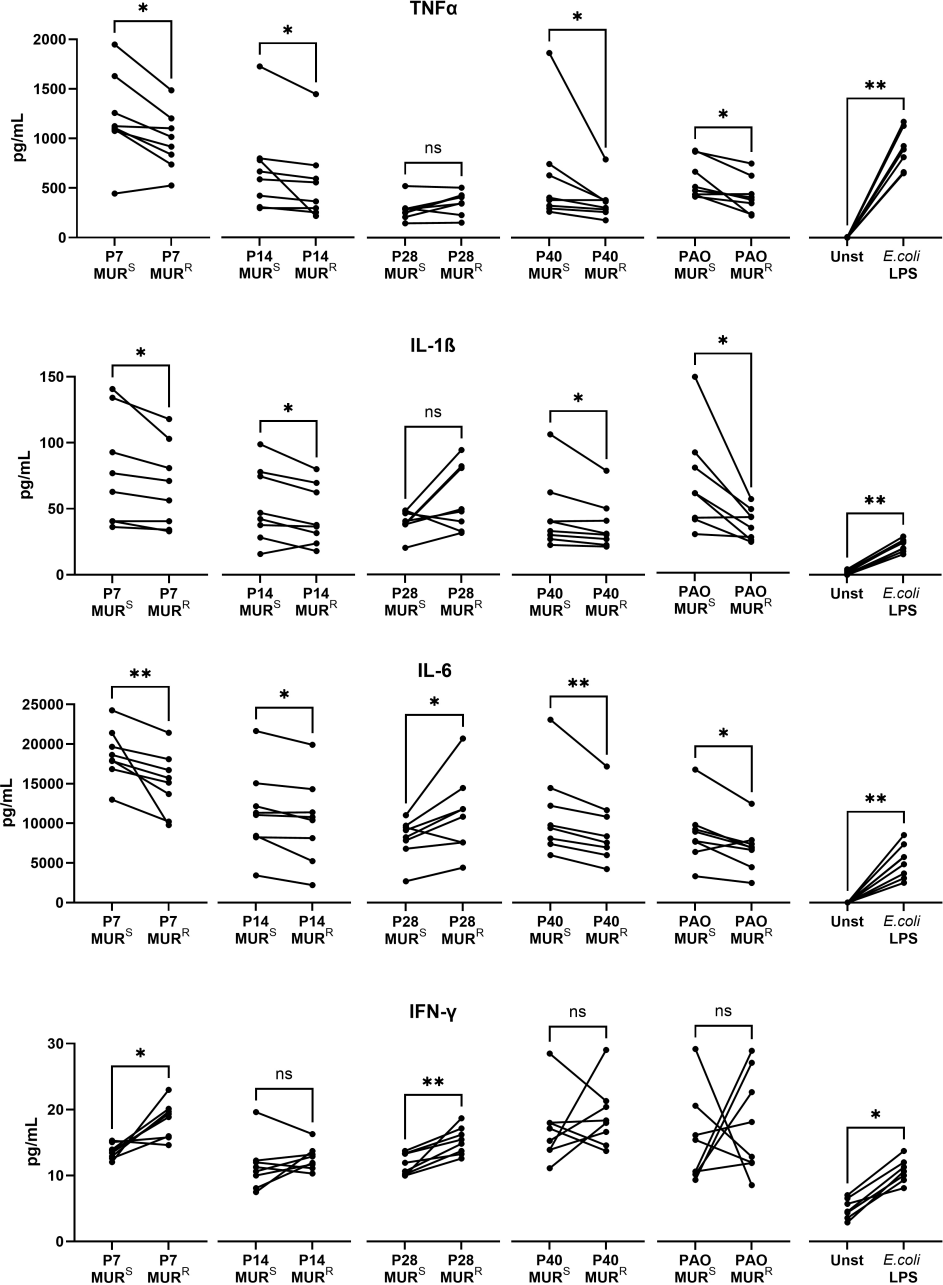
The lipopolysaccharide (LPS) from murepavadin-resistant strains (MUR^R^) exhibit lower immunogenicity than their parental murepavadin susceptible strains (MUR^S^). Human monocytes (from n=8 different healthy volunteers) were stimulated with 10 mg/L of purified LPS from MUR^R^ mutant and MUR^S^ parental strains strains for 16 h. Supernatant from unstimulated cells (Unst) and stimulated with 10 mg/L commercial *E. coli* O111:B4 LPS were used as negative and positive controls respectively. Levels of inflammatory cytokines TNFα, IL-1β, IL-6 and IFN-γ are shown. *p-value < 0.05; **p-value < 0.01; ns, no significant in Wilcoxon paired t-test.

Given that lipopolysaccharide is recognized by the immune system, the detected modifications of the murepavadin-resistant isolates could affect their inflammatory response. To measure the inflammatory response, human monocytes were stimulated with lipopolysaccharide from the 5 pairs of murepavadin-susceptible (parental) and murepavadin-resistant (mutant) isolates. The resulting murepavadin-resistant isolates appeared to induce lower expression of pro-inflammatory cytokines (tumor necrosis factor α, interleukin-1β and interleukin-6) although no clear pattern was found for interferon-γ (**Figure 4**). An exception was isolate P28, which had a small-colony-variant morphotype, and of which the resistant phenotype induced higher expression of cytokines. We confirmed that these differences were not due to apoptosis as we did not find differences when compared the apoptosis induction triggered by murepavadin-susceptible LPS to murepavadin-resistant LPS (**Supplementary Figure S3**).

### 3.5 Cross-resistance with human antimicrobial peptides

**Figure 5:**
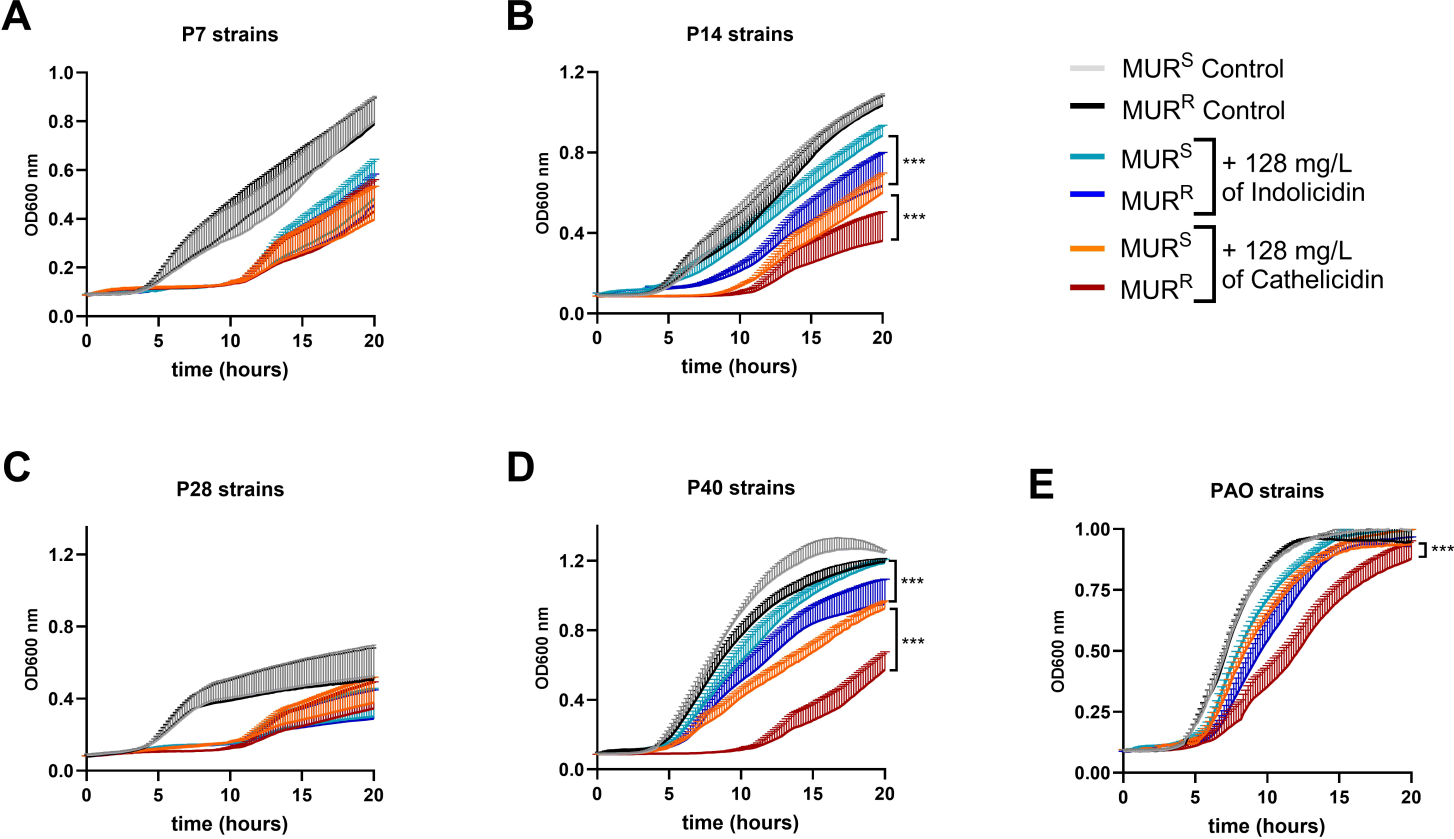
Murepavadin-resistant strains (MUR^R^) strains are more susceptible to host-AMPs than the murepavadin susceptible strains (MUR^S^). PA strains were grown in presence and absence of cathelicidin and indolicidin peptides (128 mg/L) and monitored by OD at 600 nm. **(A-E)** Growth curves of P7 **(A)**, P14 **(B)**, P28 **(C)**, P40 **(D)**, PAO1 **(E)** strains are shown. ***p-value < 0.001 in Two-way ANOVA and Tukey comparison test between murepavadin resistant mutants (MUR^R^) and murepavadin susceptible parental strains (MUR^S^) with the same treatment. Data expressed as median+SD from three independent replicates.

Growth differences between parental and mutant isolates were tested in presence of cathelicidin from human origin, and with cow neutrophil-derived indolicidin. A consistent delay in the growth of murepavadin-resistant P14 and P40 strains when exposed to human AMPs was observed when compared to their parental, murepavadin-susceptible counterparts (**Figure 5**). To note that these two strains exhibited *lpxL* mutations and a more pronounced loss of hexa-acylated lipid A forms (**Figure 3**).

## 4 Discussion

Research on peptide-derived compounds has intensified in recent years as a consequence of increasing antimicrobial resistance. Murepavadin is a cyclic peptide active against *P. aeruginosa*, including strains that produce carbapenemases and/or those that are resistant to ceftolozane/tazobactam. This antibiotic has a narrow spectrum of action as specifically target the *P. aeruginosa* LptD thought its unique N-terminal domain, and thus it is considered an environment-friendly antimicrobial (Martin-Loeches et al., 2018; Robinson, 2019). Given that its resistance mechanisms have not yet been fully elucidated, we set out to analyze genetic and phenotypic variations in resistant *P. aeruginosa* isolates, including laboratory-induced resistant mutants. We complemented this investigation by comparing the immune responses and cross-resistance to human peptides of susceptible and resistant isolates.

The prediction of resistance using WGS could be crucial for the future development of murepavadin as treatment for multidrug-resistant *P. aeruginosa*, especially in colistin-resistant isolates. In the GWAS analysis, mutations significantly associated with colistin and/or murepavadin resistance were not observed within our collection of clinical isolates from several countries and with a differential genetic background. The main limitation of our study was the limited number of colistin-resistant isolates, which are rare in our countries. Nevertheless, our data confirm that this approach is feasible, although it requires a considerably larger number of bacterial genomes to reach good levels of statistical significance. Although GWAS has been widely used to investigate genotype-phenotype association in human (Uffelmann et al., 2021), its utility is less explored in microorganisms (Saber and Shapiro, 2020).

Our GWAS analysis revealed an SNP in *hisJ* associated with murepavadin resistance. Nevertheless, despite its association, this mutation was not a direct unique causative of murepavadin-resistance as some susceptible strains also harbored it. The *hisJ* gene codifies for a periplasmic binding protein involved in histidine transport and has never been associated with antibiotic resistance. A recent study showed that *hisJ* expression could be controlled by *lasR* in *P. aeruginosa* biofilm formation (Jeske et al., 2022). Additional studies are required to define the involvement of *hisJ* in antimicrobial resistance. Overall, our GWAS results manifested that there is not a unique solely genetic variation underlying murepavadin resistance and must be a combination of several mechanisms. This is another limitation of the GWAS study, since this analysis does not allow studying the effect of the accumulation of variants on resistance phenotypes.

Mutations identified by GWAS (*hisJ*) were different from those found in our *in vitro-*derived resistant mutant strains (*lpxL1*, *lpxL2*) or in previous reports (*bamA*, *pmrB*, *msbA*) (Romano et al., 2019; Díez-Aguilar et al., 2021; Ghassani et al., 2024). This difference could be explained by two factors. First, one of the limitations of our study is that there is no established MIC cut-off value for murepavadin. We defined a tentative cut-off of 0.25 mg/L finding 23.3% of resistant strains, while Ghassani *et al*. defined it as 4 mg/L with 9.1% of resistant strains. Our isolate collection came from patients with cystic fibrosis highly exposed to antibiotics, and the expression of resistance mechanisms such as efflux pumps could not be discarded. Consequently, the epidemiological cut-off value is likely higher than that in the wild-type and thus, we could overestimate the occurrence of the resistant phenotype. Additionally, the main difference between our study and previous studies is that GWAS is an untargeted analysis that includes mutations of diverse nature (SNP, insertions or deletions) in all genomes.

Cationic AMP molecules may interact with the bacterial outer membrane; in this manner, lipopolysaccharide modifications normally reduce their antimicrobial activity (Barrow and Kwon, 2009; Jochumsen et al., 2016; Bolard et al., 2019). Isolates with the addition of the L-Ara4N motifs in lipid A are resistant to colistin (Lo Sciuto et al., 2020). Interestingly, in the murepavadin-resistant mutants, we identified a modification of lipid A compared with the parental strains. This modification was related to a lower number of acyl chains, particularly in derived isolates with mutations in *lpxL* genes. This observation was evident in strains P14 and P40. Whereas the parental strains exhibited the highest ionic peak at m/z 1632, corresponding to a hexa-acylated form of lipid A, their murepavadin-resistant mutants exhibited the penta-acylated chains m/z 1431 and 1441 as the most abundant form. Another relevant finding is that the parental isolates exhibited more abundance of the hexa-acylated lipid A form than the laboratory strain PAO1, whose main ion was the penta-acylated m/z 1447. These results agree with those of Ernst *et al*. who defined PAO1 lipid A as penta-acylated at m/z 1447 (Ernst et al., 2006). This and other studies revealed that the number of acyl chains are key mechanisms to *P. aeruginosa* adaptation in the cystic fibrosis airway (Ernst et al., 2006, 2007; Thaipisuttikul et al., 2014). These types of changes also affect immune recognition, eliciting various immune responses (Ernst et al., 2003; Pier, 2007; SenGupta et al., 2016; Avendaño-Ortiz et al., 2019). Our results indicated that the loss of hexa-acylated and subsequent increments of penta-acylated lipid A forms in murepavadin-resistant strains leads to a reduced inflammatory response. A reduction in the number of acyl chains in lipid A (e.g., from hexa-acylated to penta-acylated or tetra-acylated) has been linked to decreased stimulation of Toll-like receptor 4 (TLR4) and lower cytokine production for example in *E. coli* strains, where less acylated lipid A variants induced lower levels of inflammatory cytokines (Li et al., 2013).This outcome has also been used to explain the changes of inflammatory induction between species, e.g., the lower pro-inflammatory potency of penta-acylated *Granulibacter bethesdensis* lipid A compared with *Escherichia coli* lipid A (Lench et al., 2021). Another explanation for lower cytokine production could be the induction of cell death, such as apoptosis (Kell and Pretorius, 2015; Monguió-Tortajada et al., 2018). Since we did not find differences in LPS-induced apoptosis between resistant and susceptible strains, we can speculate that the change in the number of acyl chains might be the most plausible mechanism to explain our results in the inflammation experiments. One of the interesting aspects of our results in the inflammation experiments is that, while the main inflammatory cytokines (TNFα, IL-1β, and IL-6) decrease in the murepavadin-resistant strains compared to the susceptible ones, the production of IFN-γ were higher in two of the murepavadin-resistant strains. Consistent with our findings, it has been reported that monocytes are capable of producing low amounts of IFN-γ in response to LPS (Kraaij et al., 2014). The controversy over the source of IFN-γ production in cultures with various cell types has always existed (Darwich et al., 2009). Due to our isolation method (adherence), the basal production of IFN-γ by some Th1 T-cells that remained in the culture should not be ruled out. However, the LPS stimulation time in our experiments (16 hours) makes an antigen-specific response almost impossible, as it requires antigen presentation and several days to occur (Ulmer et al., 2000; Wölfl and Greenberg, 2014).

The number of acyl chains could also be relevant in murepavadin resistance. In the 3 strains with resistance to murepavadin that present alterations in the number of acyl chains, we observed deletions in the *lpxL1* and *lpxL2* genes. The *lpxL1* gene is responsible for adding a laurate acyl chain to the lipid A, whereas the *lpxL2* gene adds a second laurate chain at a different position. These modifications are critical for maintaining the structural integrity and functionality of the bacterial outer membrane (Hittle et al., 2015). Both *lpxL1* and *lpxL2* contribute to the biosynthesis of a hexa-acylated form of lipid A, which is essential for the bacterial membrane’s resistance to environmental stress and antimicrobial agents. Deletion or mutation of these genes can lead to altered acyl chain patterns in lipid A, affecting the bacterium’s ability to adapt to different environments (Lo Sciuto et al., 2019). Here, we found that the PAO1 strains with *lpxL1* and *lpxL2* gene deletions exhibited MICs to murepavadin that were four to eight folds higher than the parental strain. Thus, our findings supports previous studies describing the association between *lpxL1* and *lpxL2* genes mutations and murepavadin resistance (Ghassani et al., 2024).

Modifications in the lipopolysaccharide directly affect the interaction with the immune system; we previously demonstrated the tolerance of cystic fibrosis isolates after the modification of their lipopolysaccharide (Ernst et al., 2007; SenGupta et al., 2016). Herein, the murepavadin-resistant mutants reduced the number of the lipid A acyl chains, which is probably related to the downregulation of the immune response. In addition, lipid A plays a crucial role in the resistance of Gram-negative bacteria to AMPs from human origin. Understanding the mechanisms of AMP resistance has important implications for antimicrobial therapy and the development of new antimicrobial agents (Kidd et al., 2017; Assoni et al., 2020). Boll *et al*. demonstrated that the addition of an acyl palmitate chain to lipid A in *E. coli* and *Salmonella* spp. was crucial for resistance against cathelicidin (Boll et al., 2015). In addition, Nizet and Gallo discussed how modification of the bacterial wall can decrease the attractiveness of human peptides (Nizet and Gallo, 2003). The increase of acylation in lipopolysaccharide lipid A reduces the positive charge of the cell wall, and the number and length of acyl chains in lipid A also affect its physical properties (Gorman and Golovanov, 2022). Our data indicate the number of acyl chains in lipid A plays an important role in the resistance of bacteria to cathelicidin and indolicidin. Lipid A acyl chains increase bacterial resistance to peptides, while the physical properties of lipid A, influenced by the number and arrangement of acyl chains, might affect its stability and interaction with the immune system.

According to our results, murepavadin-resistant mutants with loss of hexa-acyl chains are more susceptible to cathelicidin and indolicidin, which could be crucial to achieving pathogen killing by the combined action of various AMPs. Cathelicidin is a family of diverse AMPs whose main mechanism of action is the binding to the target membrane, followed by permeabilization and/or disruption of the membrane, ultimately leading to oxidative stress and bacterial death (de Barros et al., 2019; Rowe-Magnus et al., 2019). Cathelicidin can adopt various membrane interaction models, including the carpet model, barrel model, and toroidal pore model (de Barros et al., 2019). In the case of human cathelicidin, the preform hCAP18 is processed to LL-37, which is the active antimicrobial peptide (Yamasaki et al., 2006). Indolicidin is a bovine AMP that exhibits activity against a variety of Gram-positive and Gram-negative bacteria, as well as fungi (Batista Araujo et al., 2022). Its mechanism of action involves membrane permeabilization; however, it is still not fully understood, and various models have been proposed. Indolicidin has also been shown to damage DNA, leading to an SOS response in bacteria (Rowe-Magnus et al., 2019; Vasilchenko and Rogozhin, 2019); and additionally, indolicidin inhibits DNA synthesis, leading to bacterial filamentation (Marchand et al., 2006).

In summary, using a challenging collection of clinical isolates of *P. aeruginosa* from patients with cystic fibrosis, we did not identify specific mutations associated with murepavadin resistance, alone or in combination with colistin resistance. However, in a subset of isogenic-derived resistant mutants we found significant changes in their Lipid A, mainly the loss of an acyl chain. These changes could lead to an increased immunological tolerance, but also with increased susceptibility to host AMPs.

## Data Availability

The genome datasets presented in this study can be found in online repositories. The names of the repository and accession number(s) can be found below: https://ncbi.nlm.nih.gov/bioproject/, PRJNA527751 and PRJNA530912. All the other data from the study will be provided by corresponding authors under reasonable request.
