# Supplementary material for: Unravelling the mechanisms causing murepavadin resistance in Pseudomonas aeruginosa: lipopolysaccharide alterations and its consequences
Source: Front Cell Infect Microbiol. 2024 Dec 6;14:1446626. doi: 10.3389/fcimb.2024.1446626 (PMC11659217; doi:10.3389/fcimb.2024.1446626)
Supplement: Supplementary file 1 [file DataSheet1.docx]

Supplementary Material

Unravelling the mechanisms causing murepavadin resistance in *Pseudomonas aeruginosa*: lipopolysaccharide alterations and its consequences

Marta Hernández-García^1,2†^, Raquel Barbero-Herranz^2†^, Natalia Bastón-Paz^1^, María Díez-Aguilar^3^, Eduardo López-Collazo^4,5^, Francesc J. Márquez-Garrido^6^, José María Hernández-Pérez^7^, Fernando Baquero^1,8^, Miquel B. Ekkelenkamp^9^, Ad C. Fluit^9^, Víctor Fuentes-Valverde^2,10^, Miriam Moscoso^2,10^, Germán Bou^2,10^, Rosa del Campo^1,2^, Rafael Cantón^1,2*^ José Avendaño-Ortiz^1,2*^

^1^ Servicio de Microbiología, Hospital Universitario Ramón y Cajal, and Instituto Ramón y Cajal de Investigación Sanitaria (IRYCIS), Madrid Spain.

^2^ CIBER de Enfermedades Infecciosas, Instituto de Salud Carlos III. Madrid, Spain.

^3^ Servicio de Microbiología y Parasitología, Hospital Universitario La Princesa, Madrid, Spain.

^4^ CIBER de Enfermedades Respiratorias, Instituto de Salud Carlos III. Madrid, Spain.

^5^ Innate Immune Response Group, IdiPAZ. Madrid, Spain.

^6^ Bruker Española S.A., Madrid, Spain.

^7^ Instituto de Investigación Germans Trias i Pujol, Badalona, Spain.

^8^ CIBER de Epidemiología y Salud Pública, Instituto de Salud Carlos III. Madrid, Spain.

^9^ Department of Medical Microbiology, University Medical Center Utrecht, Utrecht, The Netherlands.

^10^ Department of Microbiology, University Hospital A Coruña (CHUAC)-Biomedical Research Institute A Coruña (INIBIC), 15006 A Coruña, Spain.

†These authors share first authorship

*** Correspondence** (These authors share last authorship): Rafael Cantón ([rafael.canton@salud.madrid.org](mailto:rafael.canton@salud.madrid.org)), and José Avendaño-Ortiz ([joseavenort@gmail.com](mailto:joseavenort@gmail.com)).

**SUPPLEMENTARY FIGURES**

**
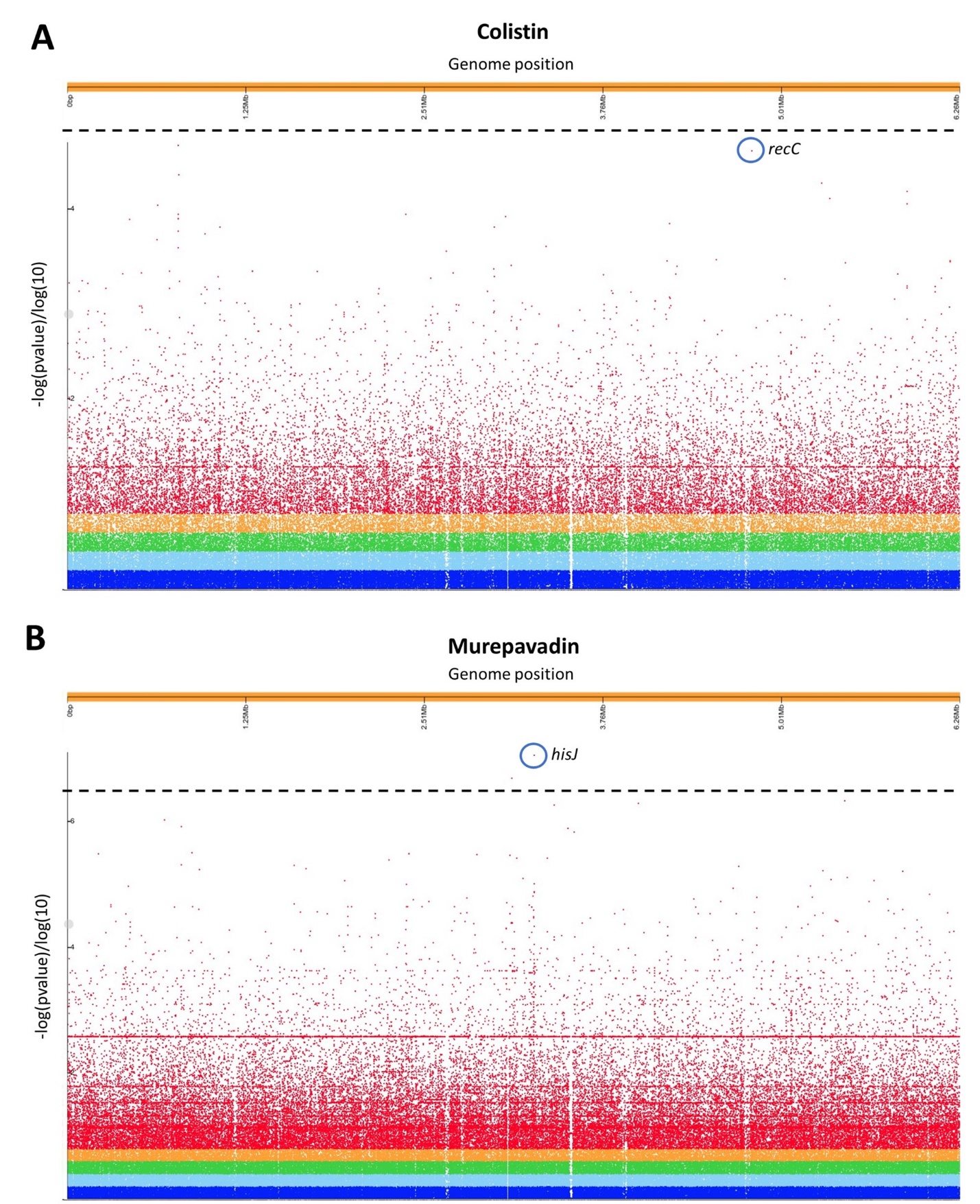
**

**Supplementary figure 1.** Manhattan plots of GWAS for colistin (**A**) and murepavadin (**B**) resistance plotted by Phandango. Each dot is the allele variant tested (n=102,008) and their position in the X axis is related to the reference genome of PAO1 (the orange line in the upper part of each plot represent the size of the whole genome) and their position on the Y axis constitutes the corrected *p*-value (LRT_*p*) for resistant phenotype in the population structured as [y=-log(LRT_*p*)/log(10)]. Circled dots correspond with the highest significant SNPs in known genes in each plot. Dotted black line represent the statistically significance threshold from the Bonferroni correction method (*p*=4.9 x10^-7^).


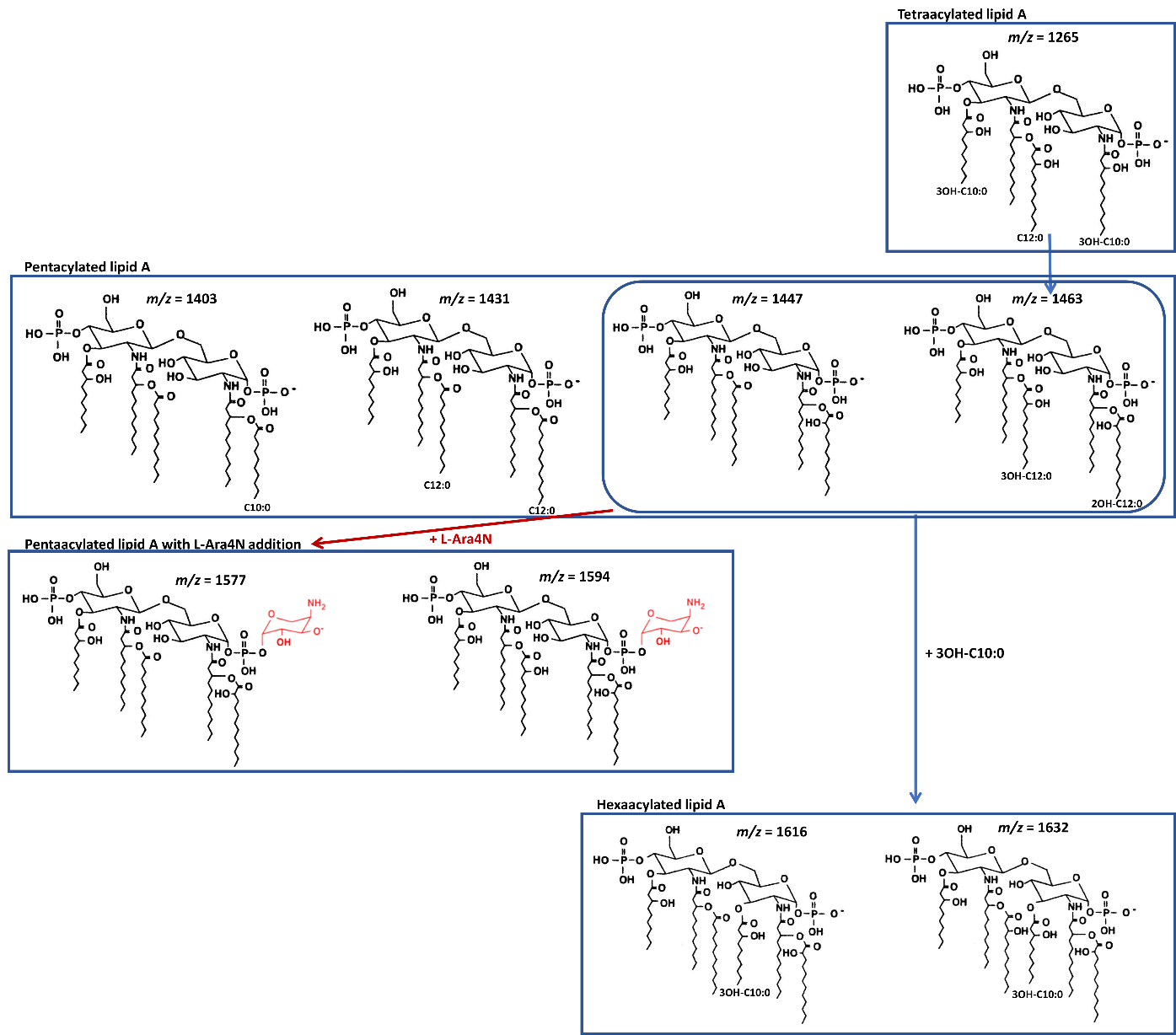


**
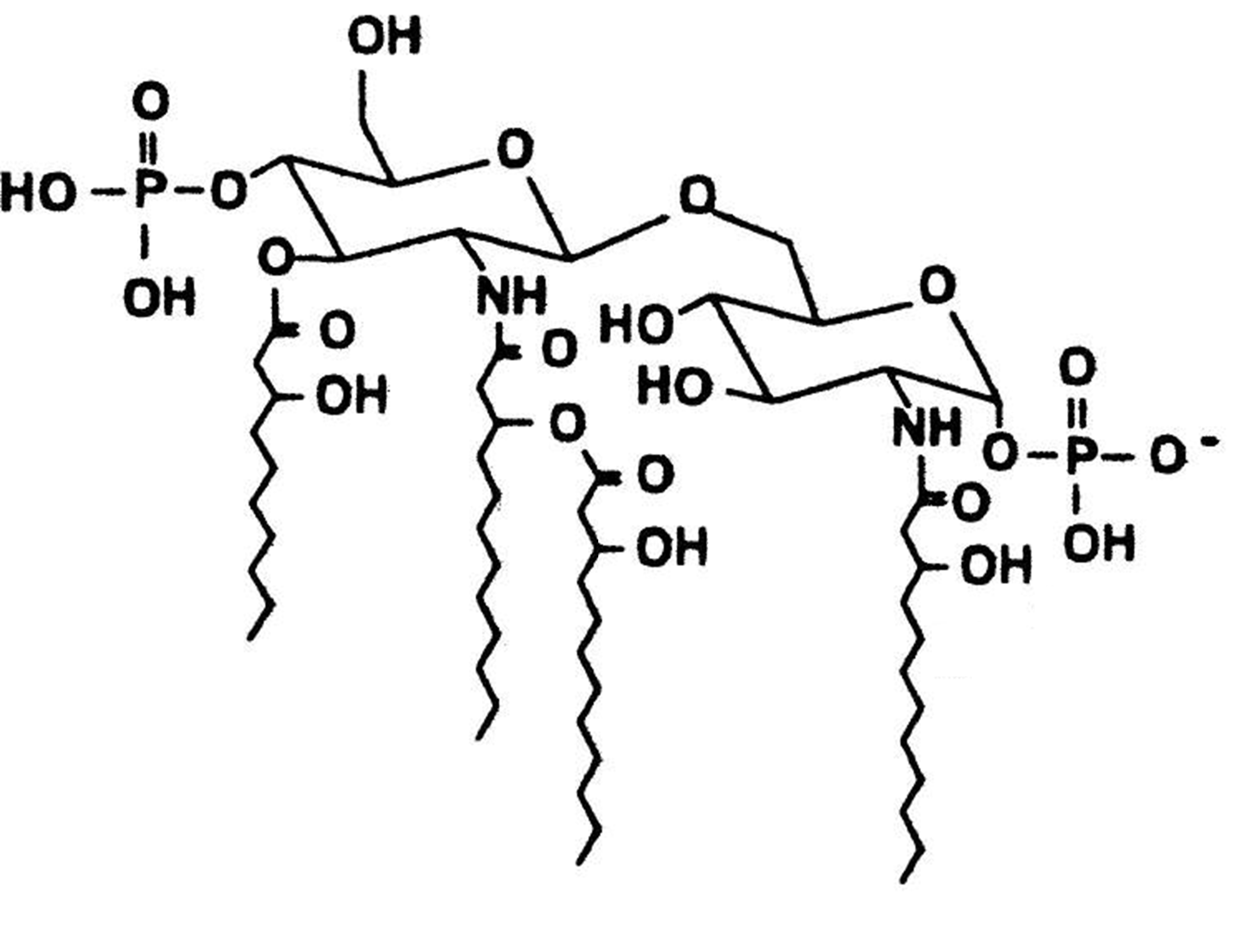

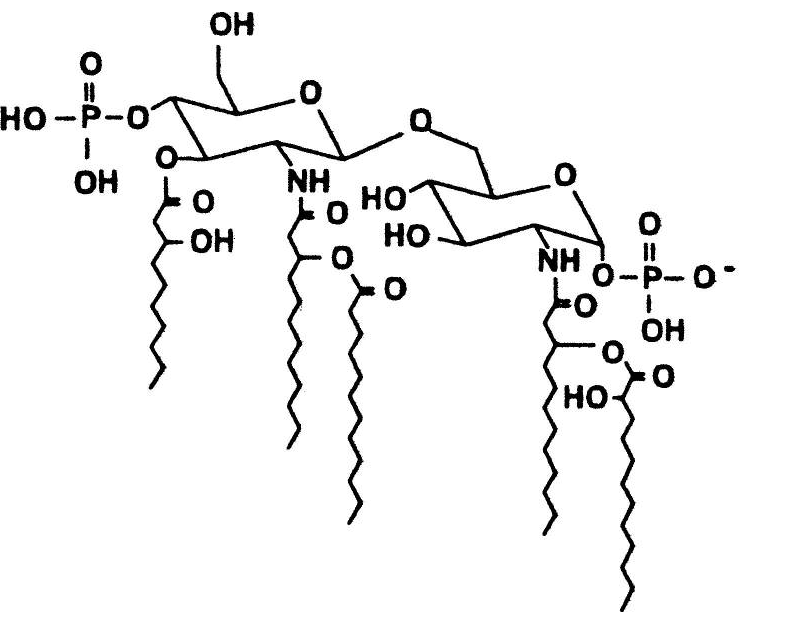

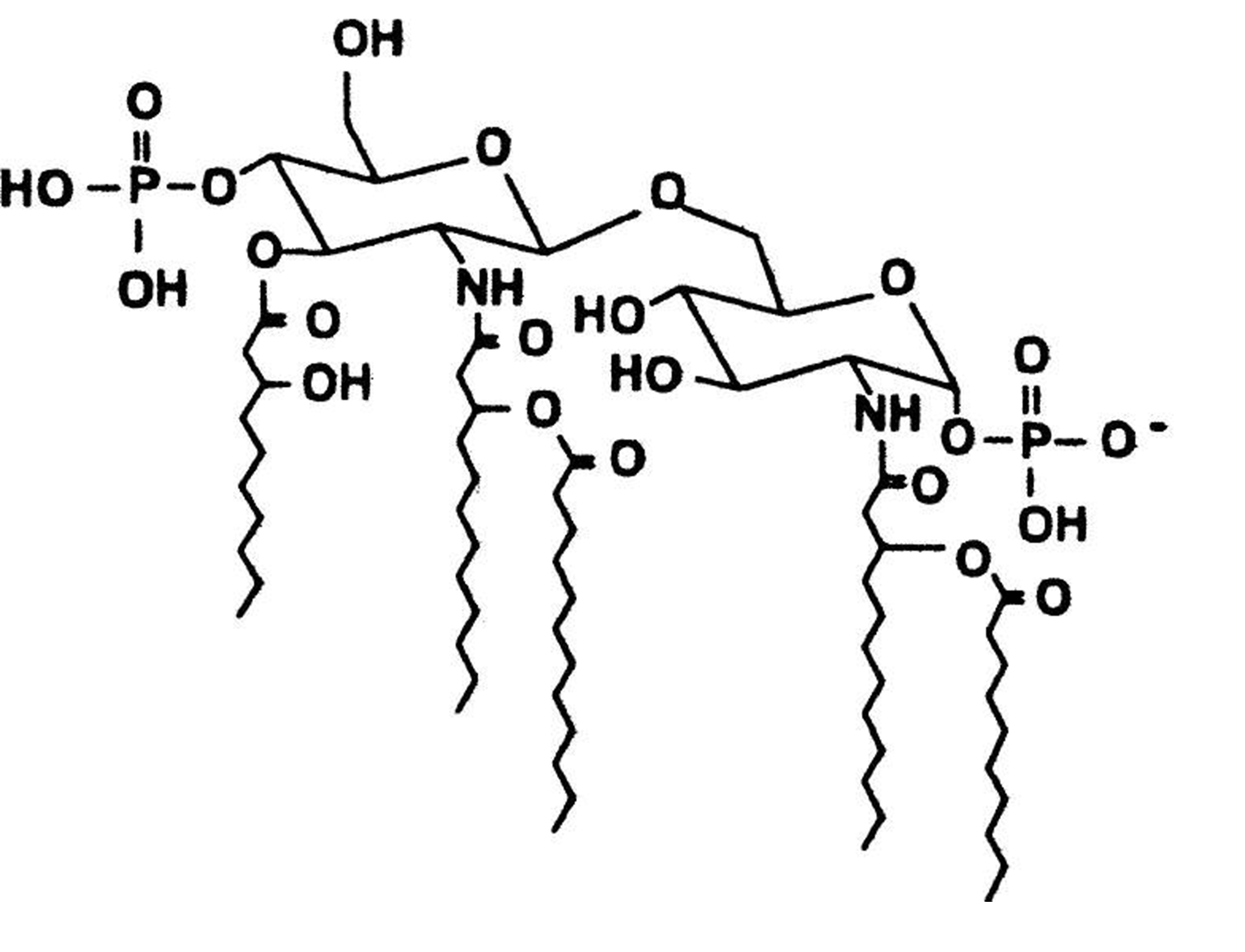

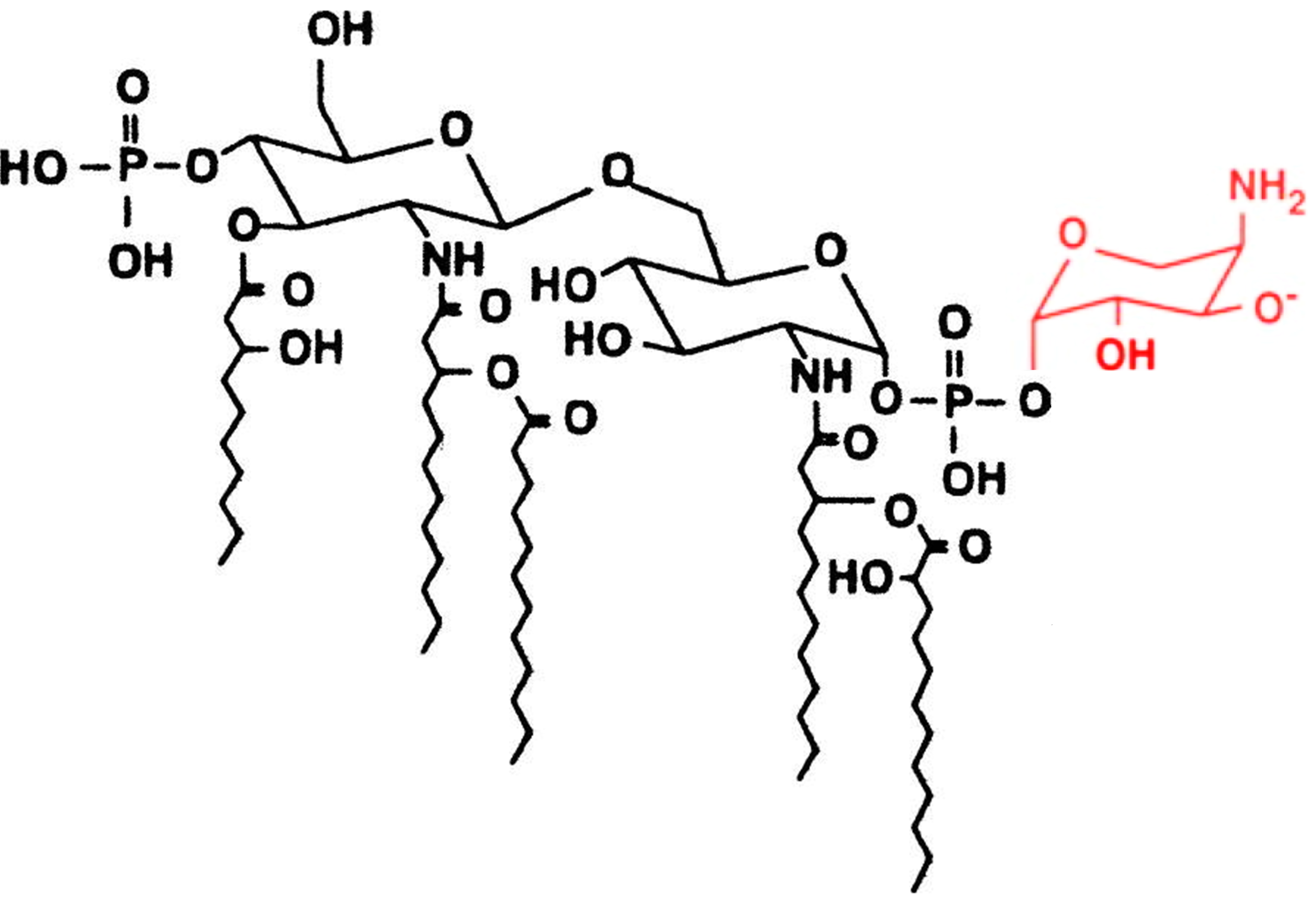

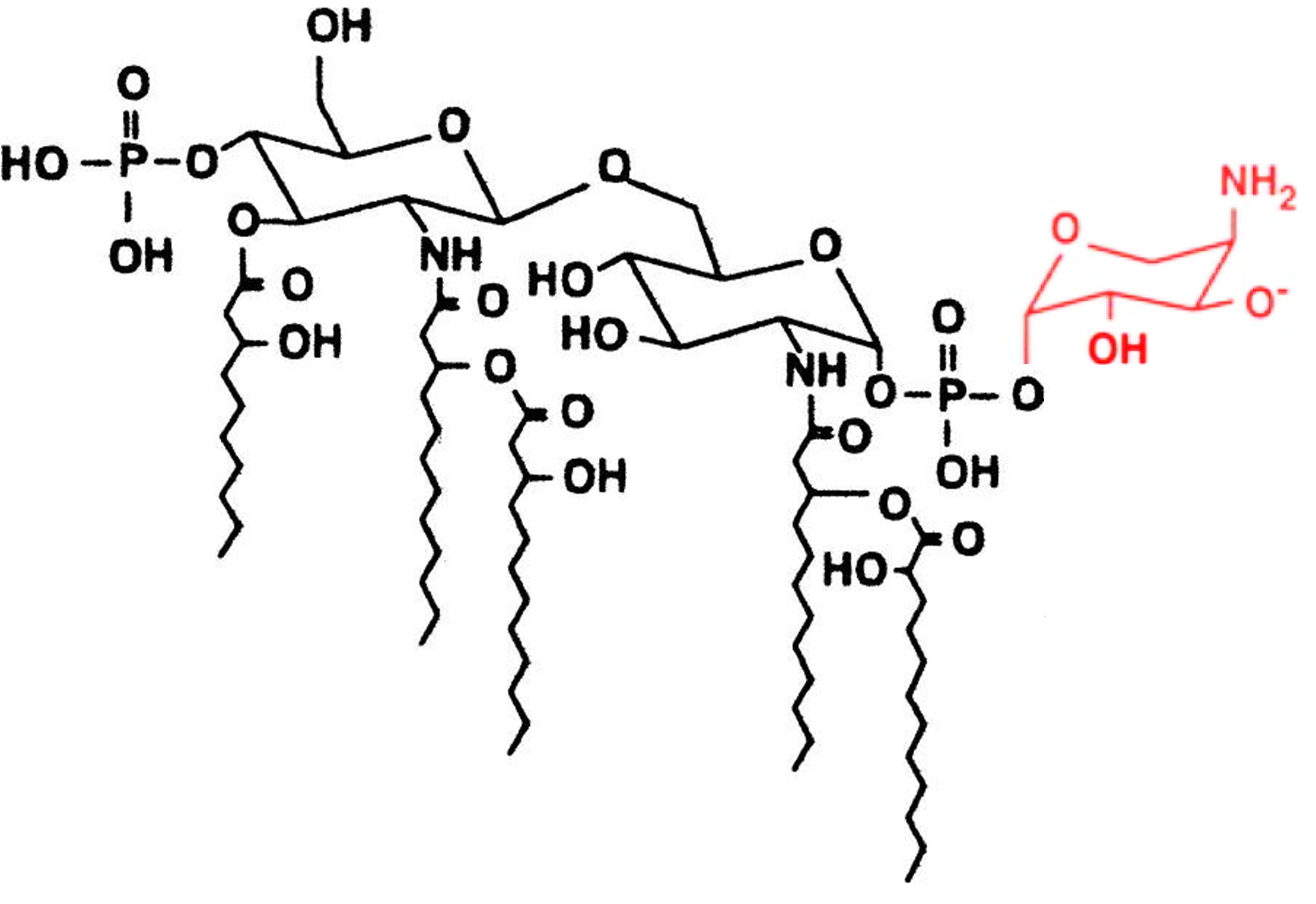

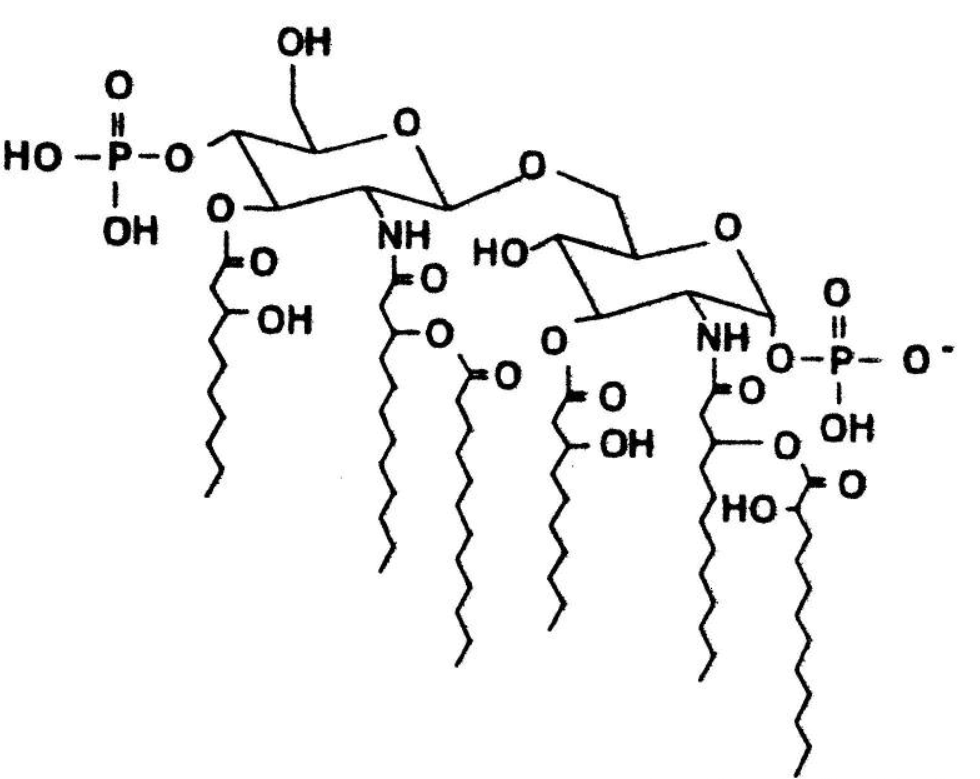

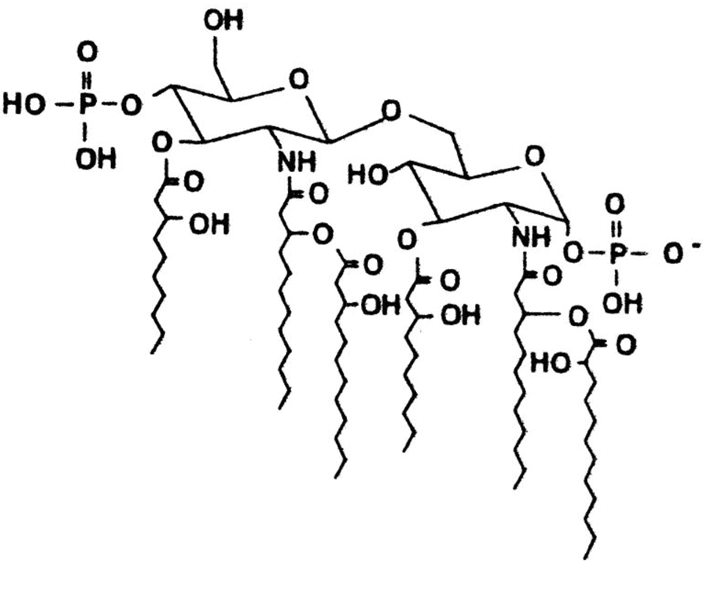

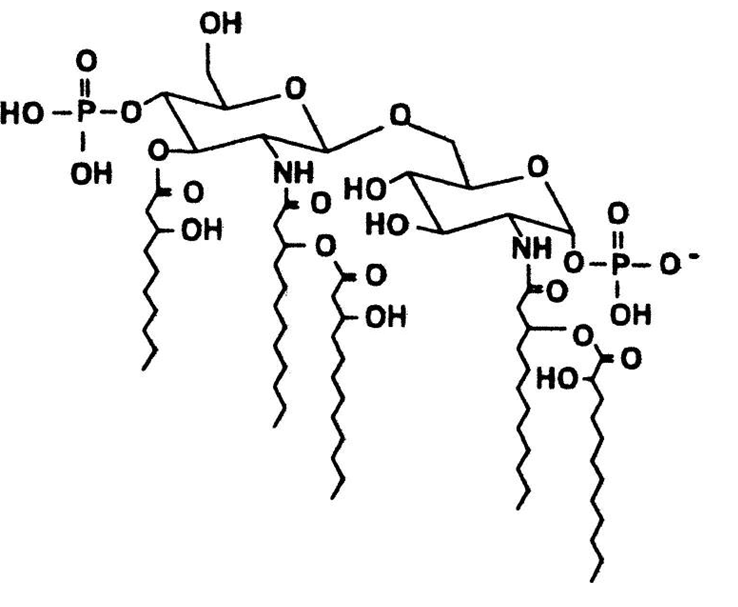
Supplementary figure 2**. Proposed lipid A structures for the lipid A ion peaks identified in MALDI-TOF spectra analysis.

***m/z* = 1616**

**Hexaacylated lipid A**

***m/z* = 1431**

***m/z* = 1447**

***m/z* = 1577**

**Pentaacylated lipid A with L-Ara4N addition**

***m/z* = 1632**

***m/z* = 1463**

***m/z* = 1403**

***m/z* = 1265**

***m/z* = 1594**

**+ L-Ara4N**

**3OH-C10:0**

**C12:0**

**3OH-C10:0**

**3OH-C12:0**

**2OH-C12:0**

**3OH-C10:0**

**3OH-C10:0**

**Pentacylated lipid A**

**Tetraacylated lipid A**

**+ 3OH-C10:0**

**
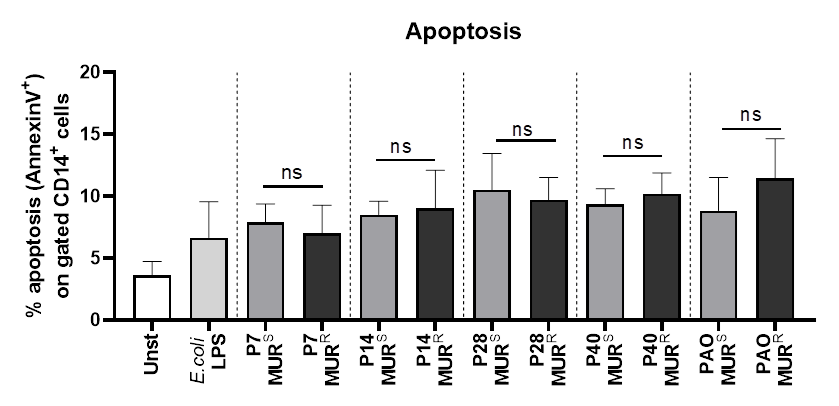
**

**
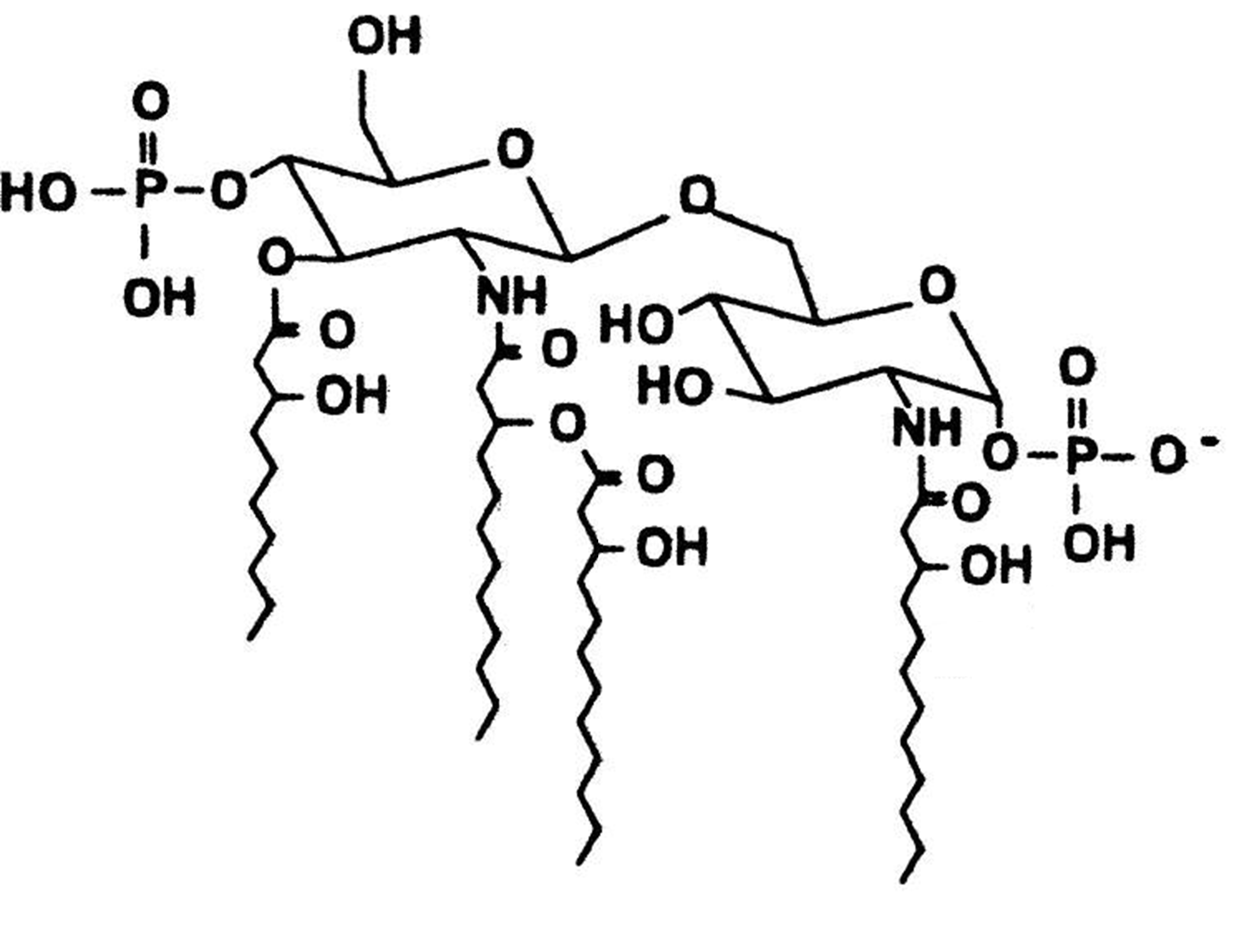

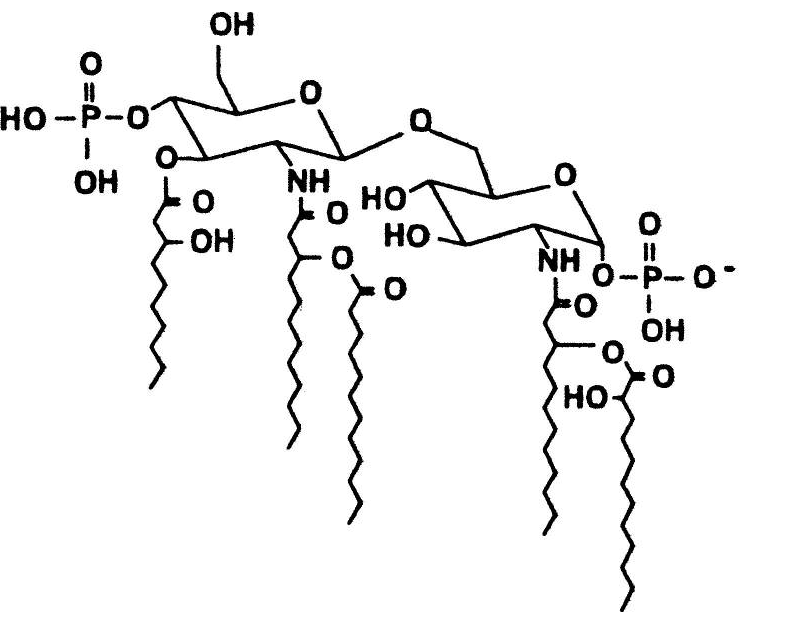

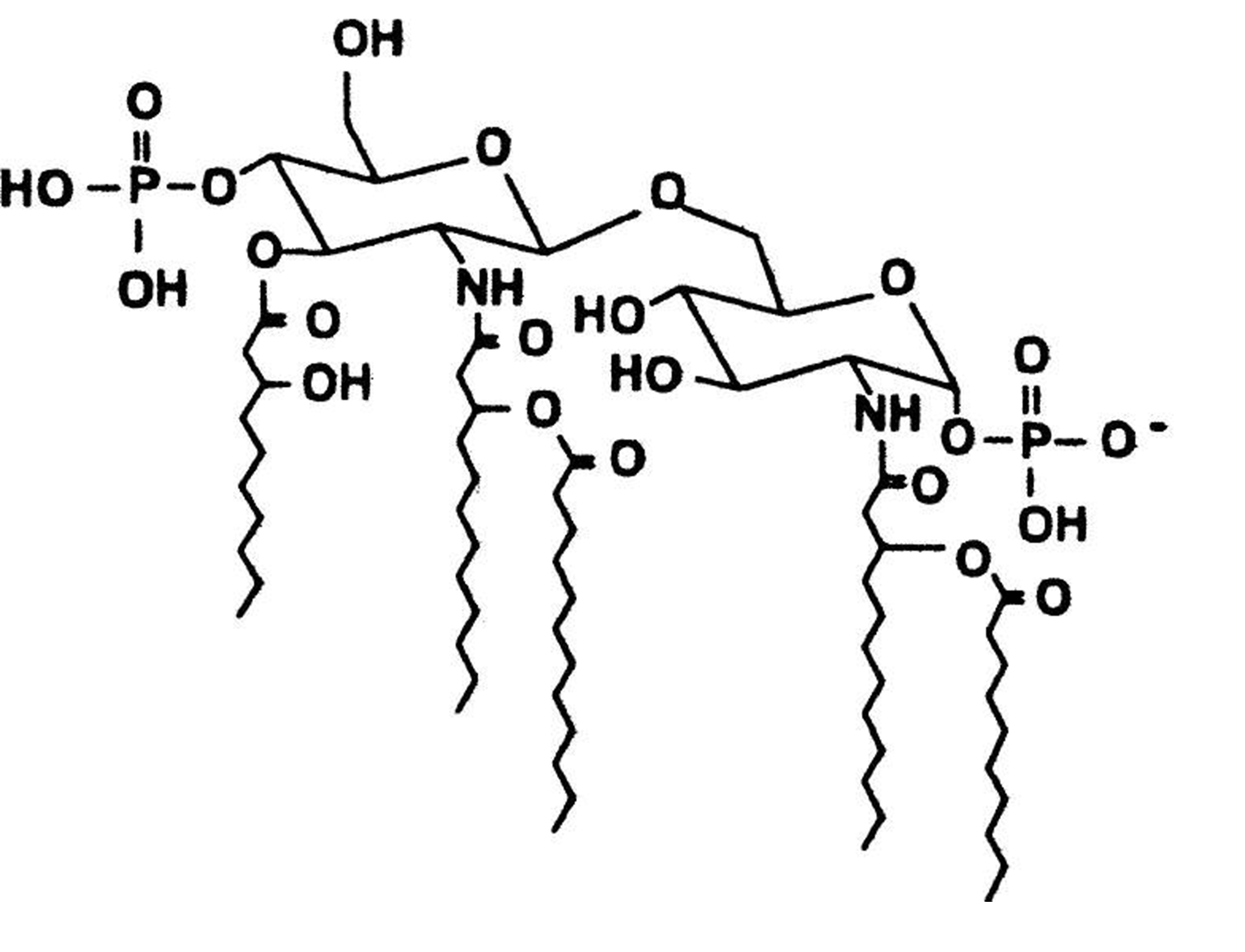

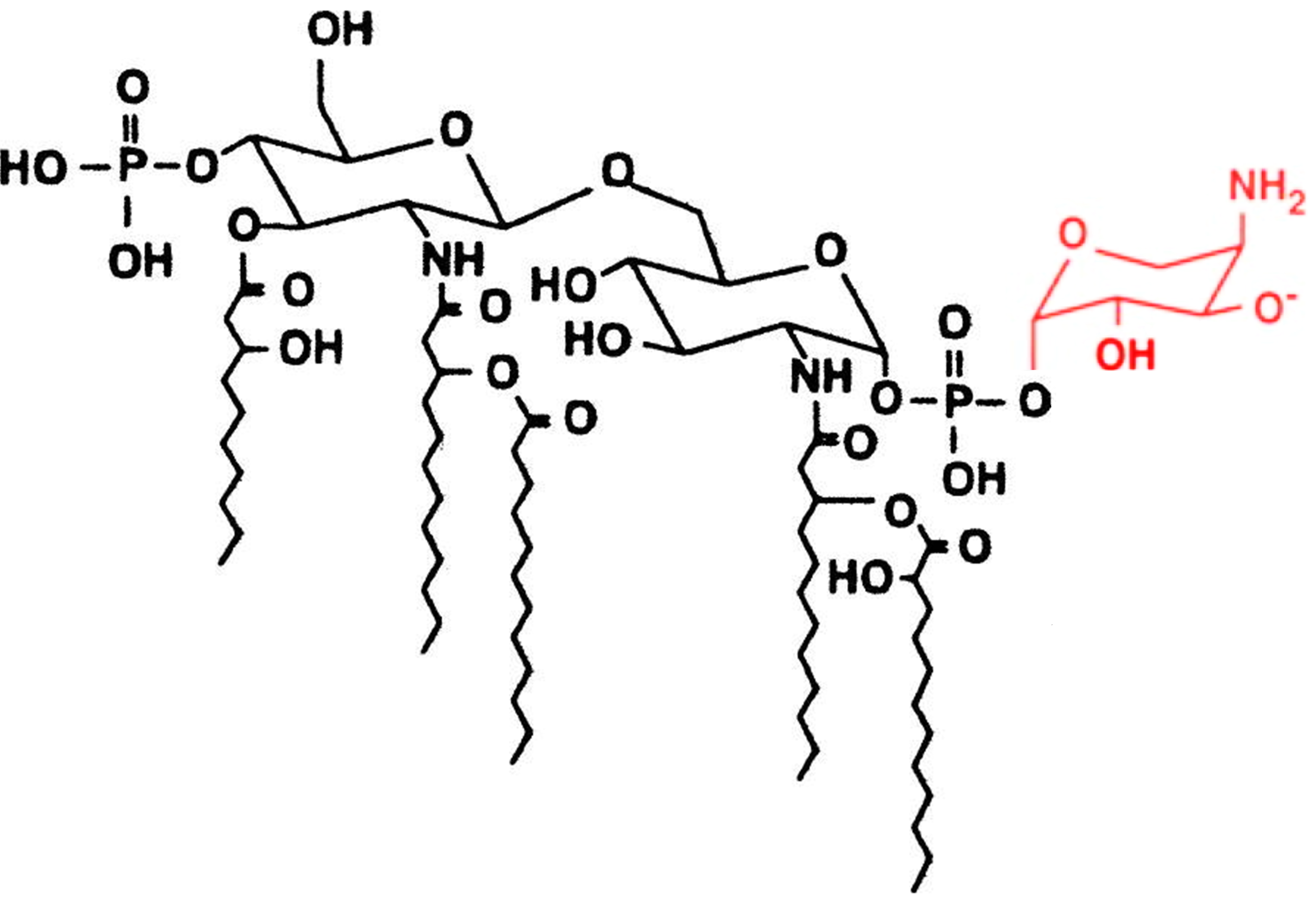

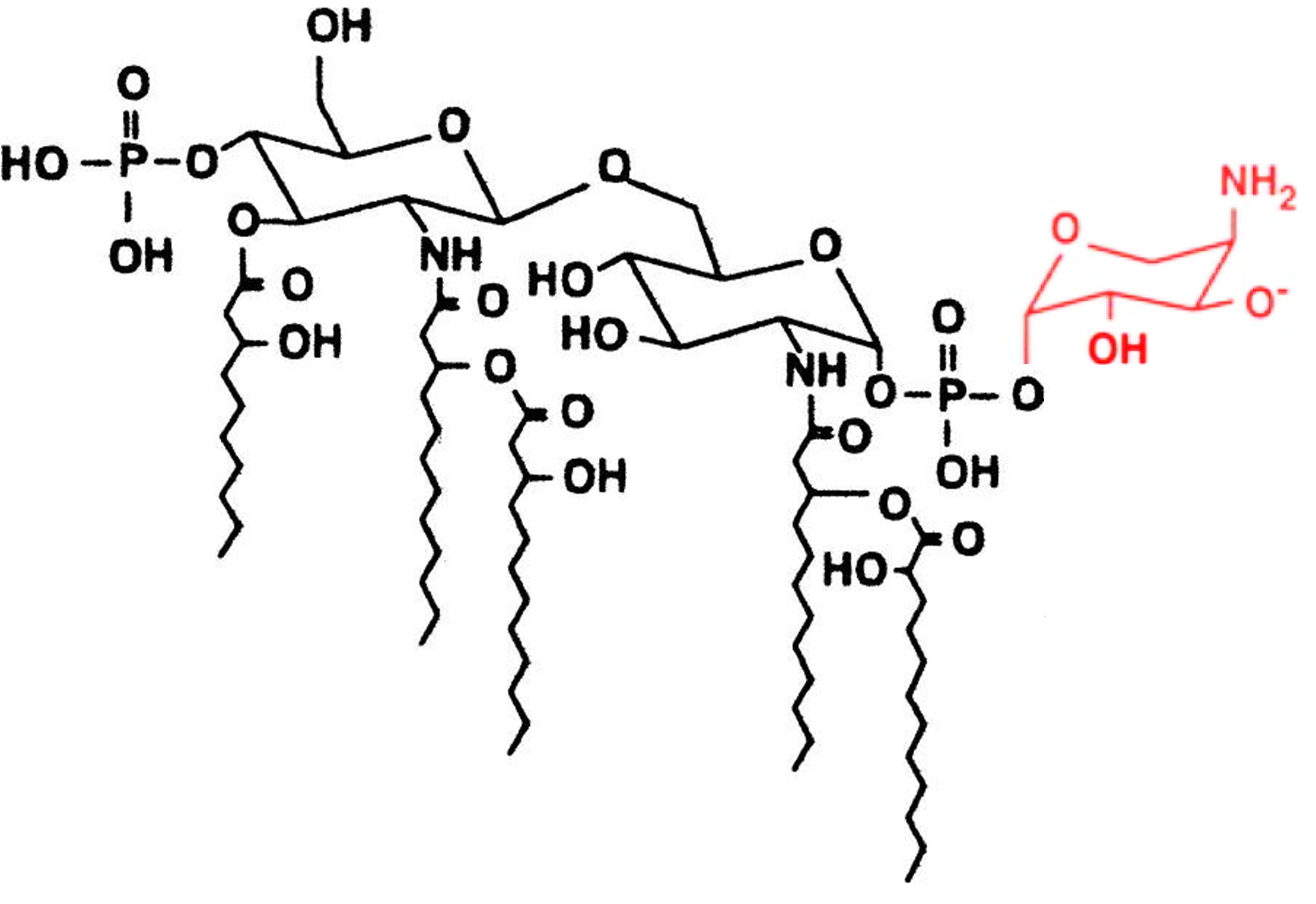

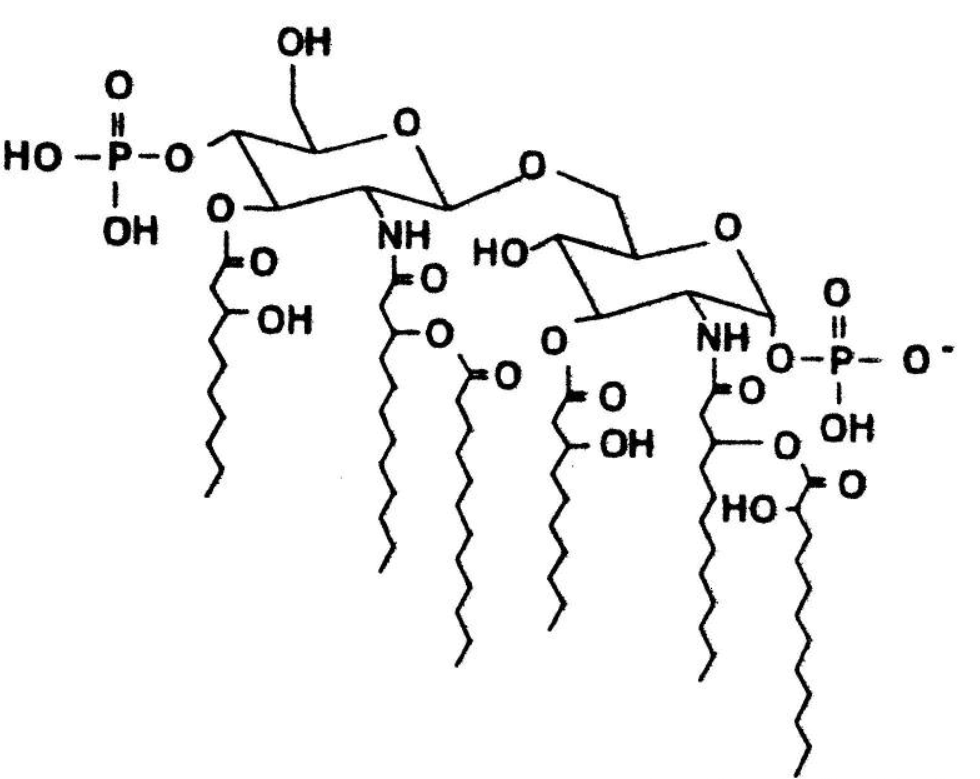

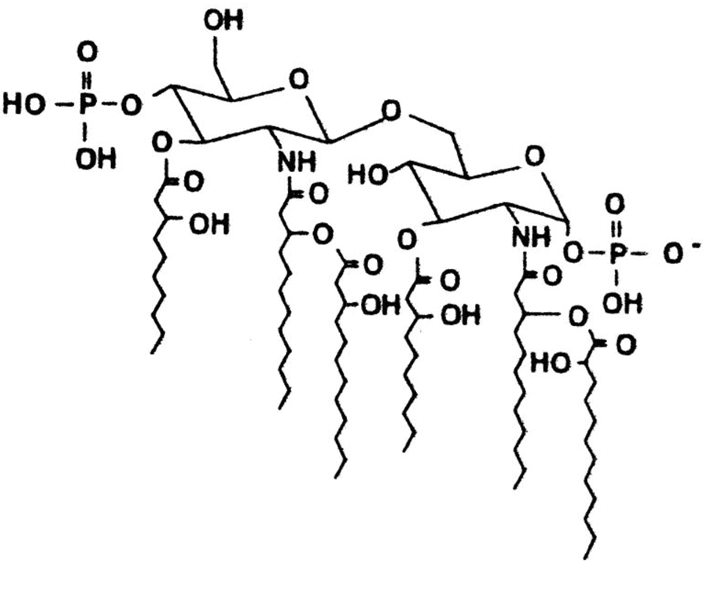

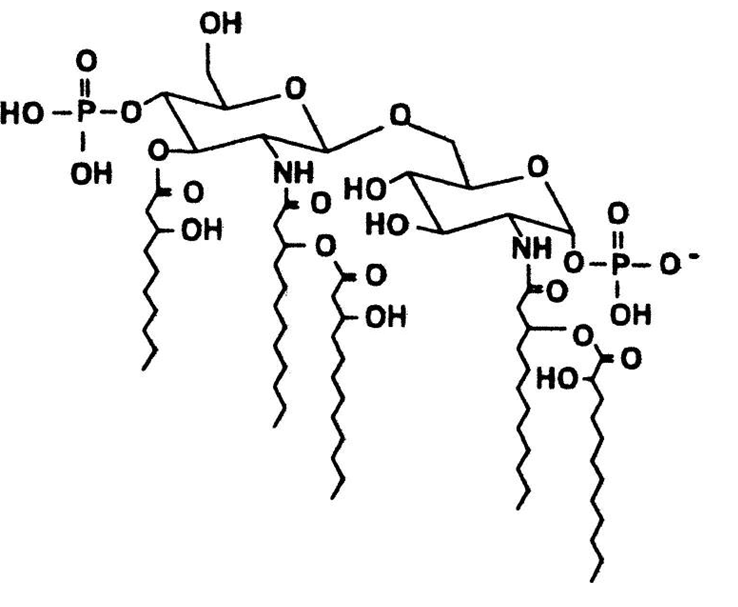
Supplementary figure 3**. Apoptosis of monocytes stimulated with LPS from LPS from murepavadin resistant mutants (MUR^R^) and murepavadin susceptible parental strains (MUR^S^) strains for 16 h.. Human monocytes (from n=4 different healthy volunteers) were stimulated with 10 ng/mL of purified LPS for 16 h. Unstimulated cells (Unst) and stimulated with 10 ng/mL commercial *E. coli* O111:B4 LPS were used as negative and positive controls respectively. Apoptosis defined as Annexin V+ cells on gated CD14+ cells are shown. ns, no significant in Wilcoxon paired t-test; Unst, unstimulated cell.

***m/z* = 1616**

**Hexaacylated lipid A**

***m/z* = 1431**

***m/z* = 1447**

***m/z* = 1577**

**Pentaacylated lipid A with L-Ara4N addition**

***m/z* = 1632**

***m/z* = 1463**

***m/z* = 1403**

***m/z* = 1265**

***m/z* = 1594**

**+ L-Ara4N**

**3OH-C10:0**

**C12:0**

**3OH-C10:0**

**3OH-C12:0**

**2OH-C12:0**

**3OH-C10:0**

**3OH-C10:0**

**Pentacylated lipid A**

**Tetraacylated lipid A**

**+ 3OH-C10:0**

**SUPPLEMENTARY TABLES**

**Supplementary table 1.** Isolates used in lipid A analysis. In bold are marked those MIC values considered as resistant (murepavadin MIC >0.25 mg/L and colistin MIC >4 mg/L).

| Isolate | Murepavadin MIC (mg/L) | Colistin MIC (mg/L) | Colony variant phenotype | MLST |
| --- | --- | --- | --- | --- |
| P7 | **2** | **16** | Non-mucoid | ST497-1LV |
| P7-mutant | **>16** | **16** |  |  |
|  |  |  |  |  |
| P14 | 0.12 | 1 | Non-mucoid | ST535-1LV |
| P14-mutant | **>16** | 1 |  |  |
| P28 | 0.12 | 0.25 | Small colony variant | ST569 |
| P28-mutant | **4** | 0.25 |  |  |
|  |  |  |  |  |
| P40 | 0.06 | 1 | Non-mucoid | ST379-1LV |
| P40-mutant | **4** | 2 | Mucoid |  |
|  |  |  |  |  |
| PAO1 | 0.06 | 1 | Non-mucoid | ST549 |
| PAO1-mutant | **1** | 1 |  |  |

**Supplementary table 2.** Allelic variants with higher association to colistin resistant phenotype in the GWAS analysis comparing 32 colistin-resistant *versus* 464 colistin-susceptible. For a complete list of SNPs, see the excel file Supplementary document 2.

| **Gene** | **Position in the genome** | **variant** | **LRT *p-*value** | **β effect size** | **OR** |
| --- | --- | --- | --- | --- | --- |
| Not annotated region | 782998 | G→A | 1.97x10^-5^ | 4.57 | 95.5 |
| *recC* | 4805950 | C→T missense variant (Asp→Asn) | 2.25x10^-5^ | 3.16 | 23.1 |
| Not annotated region | 3077286 | C→T | 1.12x10^-4^ | 2.62 | 13.7 |
| Hypothetical protein | 4229701 | G→A missense variant (Ala→Thr) | 1.33x10^-4^ | 2.71 | 15.0 |
| Not annotated region (close to *gloA* gene) | 782564 | GTTTC→ATTTT | 1.60x10^-4^ | 3.36 | 28.8 |
| *lgrD* | 4556795 | CCTGT→TCTGG missense variant (Ser→Ala) | 3.21x10^-4^ | 5.88 | 357.8 |

OR, odds ratio; LRT, Likelihood Ratio Test.

**Supplementary table 3.** Allelic variants with higher association to murepavadin resistant phenotype in the GWAS analysis comparing 133 murepavadin-resistant *versus* 363 murepavadin-susceptible isolates. For a complete list of SNPs, see excel file Supplementary document 3.

| **Gene** | **Position in the genome** | **variant** | **LRT *p-v*alue** | **β effect size** | **OR** |
| --- | --- | --- | --- | --- | --- |
| *hisJ* | 3279415 | A→G missense variant (Thr→Ala) | 7.77x10^-8^ | 2.64 | 14.3 |
| Not annotated region (close to *oprD* gene) | 3119884 | G→A | 1.81x10^-7^ | 4.5 | 88.2 |
| Not annotated region | 5457738 | GC→G deletion | 4.17x10^-7^ | 3.59 | 35.5 |
| Hypothetical protein | 685581 | GCGC→CGT frameshift variant/missense variant (Arg→fs) | 8.44x10^-7^ | 3.43 | 34 |
| *rcsC_4* | 2399248 | GGAAA→CGAAG missense variant (Ser→Gly) | 3.19x10^-6^ | 5.81 | 333.6 |
| *rcsC_4* | 2399258 | CGTA→AGGG missense variant (Thr→Ala) | 3.19x10^-6^ | 5.81 | 333.6 |

OR, odds ratio; LRT, Likelihood Ratio Test.

**Supplementary table 4.** Allelic variants associated with both colistin and murepavadin resistance comparing the double resistant isolates (n=17) *versus* susceptible to both antibiotics (n=349). For a complete list of SNPs, see excel file Supplementary document 4.

| **Gene** | **Position in the genome** | **variant** | **LRT_*p*-value** | **β effect size** | **OR** |
| --- | --- | --- | --- | --- | --- |
| intergenic region | 3216365 | G→A | 2.52x10^-5^ | 6.44 | 225.88 |
| hypothetical protein | 3422754 | C→G missense variant (Val→Leu) | 2.76x10^-5^ | 3.41 | 22.87 |
| intergenic region | 3825869 | C→A | 3.44x10^-5^ | 2.64 | 15.3 |
| intergenic region | 1133142 | A→T | 6.63x10^-5^ | 2.7 | 16.9 |
| *YhjE* | 4201792 | G→A missense variant (Ala→Thr) | 7.76x10^-5^ | 2.92 | 18.36 |
| *YofA* | 2944454 | T→C missense variant (Asp→Gly) | 7.82x10^-5^ | -2.56 | 0.07 |

OR, odds ratio; LRT, Likelihood Ratio Test.
